# Sex in the shadow of HIV: A systematic review of prevalence, risk factors, and interventions to reduce sexual risk-taking among HIV-positive adolescents and youth in sub-Saharan Africa

**DOI:** 10.1371/journal.pone.0178106

**Published:** 2017-06-05

**Authors:** Elona Toska, Marija Pantelic, Franziska Meinck, Katharina Keck, Roxanna Haghighat, Lucie Cluver

**Affiliations:** 1 Department of Social Policy and Intervention, University of Oxford, Oxford, United Kingdom; 2 AIDS and Society Research Unit, Centre for Social Science Research, University of Cape Town, Cape Town, South Africa; 3 OPTENTIA, School of Behavioural Sciences, North-West University, Vanderbeijlpark, South Africa; 4 Oxford Policy Management, Johannesburg, South Africa; 5 Department of Psychiatry and Mental Health, University of Cape Town, Cape Town, South Africa; ARGENTINA

## Abstract

**Background:**

Evidence on sexual risk-taking among HIV-positive adolescents and youth in sub-Saharan Africa is urgently needed. This systematic review synthesizes the extant research on prevalence, factors associated with, and interventions to reduce sexual risk-taking among HIV-positive adolescents and youth in sub-Saharan Africa.

**Methods:**

Studies were located through electronic databases, grey literature, reference harvesting, and contact with researchers. Preferred Reporting Items for Systematic Reviews and Meta-Analyses guidelines were followed. Quantitative studies that reported on HIV-positive participants (10–24 year olds), included data on at least one of eight outcomes (early sexual debut, inconsistent condom use, older partner, transactional sex, multiple sexual partners, sex while intoxicated, sexually transmitted infections, and pregnancy), and were conducted in sub-Saharan Africa were included. Two authors piloted all processes, screened studies, extracted data independently, and resolved any discrepancies. Due to variance in reported rates and factors associated with sexual risk-taking, meta-analyses were not conducted.

**Results:**

610 potentially relevant titles/abstracts resulted in the full text review of 251 records. Forty-two records (n = 35 studies) reported one or multiple sexual practices for 13,536 HIV-positive adolescents/youth from 13 sub-Saharan African countries. Seventeen cross-sectional studies reported on individual, relationship, family, structural, and HIV-related factors associated with sexual risk-taking. However, the majority of the findings were inconsistent across studies, and most studies scored <50% in the quality checklist. Living with a partner, living alone, gender-based violence, food insecurity, and employment were correlated with increased sexual risk-taking, while knowledge of own HIV-positive status and accessing HIV support groups were associated with reduced sexual risk-taking. Of the four intervention studies (three RCTs), three evaluated group-based interventions, and one evaluated an individual-focused combination intervention. Three of the interventions were effective at reducing sexual risk-taking, with one reporting no difference between the intervention and control groups.

**Conclusion:**

Sexual risk-taking among HIV-positive adolescents and youth is high, with inconclusive evidence on potential determinants. Few known studies test secondary HIV-prevention interventions for HIV-positive youth. Effective and feasible low-cost interventions to reduce risk are urgently needed for this group.

## Introduction

With increased access to antiretroviral treatment in sub-Saharan Africa, the number of children vertically infected with HIV who survive to adolescence has risen [1,2]. Coupled with sustained high HIV-incidence among youth in the region, this has resulted in nearly 1.7 million HIV-positive adolescents (10–19 years old) in sub-Saharan Africa, with girls representing nearly two-thirds of this total [3–5]. Despite global reductions in HIV prevalence, rates of new HIV infections remain the highest among 15–24 year old youth in sub-Saharan Africa [6]. As their numbers continue to grow, adolescents and youth living with HIV are an essential group for secondary HIV prevention efforts [7].

HIV-positive adolescents and youth are at risk of passing on the virus to their sexual partners and children [8,9]. They are additionally vulnerable to potential re-infection by HIV and more vulnerable to other sexually transmitted infections (STIs) compared to their HIV-negative peers [10]. Adolescents are more likely than adults or younger children to adhere poorly to their medication [11–13] and in particular to treatment regimens to prevent mother-to-child-transmission [14]. Low adherence and retention in care rates are strongly associated with resistance to available antiretroviral therapies, including second-line treatment when available [15,16]. With limited access to second and third-line antiretroviral treatment, HIV-positive adolescents risk running out of treatment options or infecting others with resistant strains of the virus. In addition, HIV-positive adolescents experience a range of vulnerabilities that reduce the efficacy of generalised prevention programmes, including cognitive and mental health issues [17,18], family-related challenges [19,20], and material deprivation [21,22]. Adolescents living with HIV in sub-Saharan Africa are particularly vulnerable to these risks due to poor access to healthcare services such as family planning, HIV testing, and treatment [23–27].

A small number of studies on adolescents living with HIV in sub-Saharan Africa report high rates of unprotected sex [28–30]; however, little is known about rates of other high-risk practices, such as transactional sex, sex with older partners, and multiple concurrent sexual partners [31]. In the general adolescent population, these high-risk sexual practices have been associated with higher odds of becoming infected with HIV [32]. Though the evidence on different high-risk sexual practices among HIV-positive adolescents is nascent, understanding factors associated with sexual risk-taking is crucial for intervention development.

Although some interventions to reduce sexual risk behaviours have been conducted among HIV-positive adolescents in the United States [33–37], there is a dearth of research and interventions on secondary prevention among HIV-positive adolescents in the developing world [38]. A 2010 WHO review of behavioural interventions for HIV positive prevention in middle and lower-income countries found 19 studies, none of which focused on young people [39]. A recent review of sexual and reproductive health and rights interventions for youth living with HIV in sub-Saharan Africa located six small-scale interventions [38], only three of which quantitatively measured change in a sexual risk behaviour [40–42].

To fill the evidence gap in effective interventions for this vulnerable population, further research is needed to elucidate HIV-positive adolescent sexual and reproductive health needs. This includes a better understanding of the epidemiology of sexual risk-taking as well as hypothesized models of sexual health decision-making among HIV-positive adolescents and youth [43]. This systematic review synthesizes existing evidence of sexual risk-taking among HIV-positive adolescents and youth in sub-Saharan Africa (10–24 years old) on: 1) prevalence 2) factors associated with of risk taking, and 3) interventions.

## Methods

This review follows the Preferred Reporting Items for Systematic Reviews and Meta-Analyses (PRISMA) guidelines [44]. The scope of this review (Table 1) is to assess the state of the evidence for three research questions:

1. What is the prevalence of sexual risk-taking among HIV-positive adolescents and youth in sub-Saharan Africa?
2. What factors (correlates, risk factors, or predictors) are associated with sexual risk-taking among HIV-positive adolescents and youth in sub-Saharan Africa?
3. Which interventions, aimed at reducing sexual risk-taking among HIV-positive adolescents and youth in sub-Saharan Africa, have been tested, and how effective were they?

**Table 1:** Scope of systematic review.

*Population*	Adolescents and Youth living with HIV Age range: 10–24 years old
*Outcome*	Individual risk behaviours: early sexual debut, unprotected sex (inconsistent condom use/ contraception use), having an older partner, transactional sex, having multiple sexual partners, sex drunk or on drugs, sexually transmitted infections, and unintended adolescent pregnancies. OR Composite risk behaviours consisting of any of the above behaviours combined.
*Geographic location*	sub-Saharan Africa
*Study Design*	Randomised controlled trials (individual or cluster), Quasi-experimental studies including quasi-randomized trials, controlled before-after studies, pre- and post-test studies, longitudinal cohort studies, cross-sectional studies

**Inclusion criteria** applied consisted of study population, design, sampling strategy, outcome measures, population type, and language (S1 Table). To document outcome prevalence and factors associated with the outcomes, cross-sectional surveys and longitudinal prospective cohort studies were included. Although Randomised Controlled Trials (RCT) provide the strongest form of evidence about intervention impact, due to the small number of RCTs identified in preliminary searches, this review also included studies with less rigorous designs: pre-post intervention comparisons and post-intervention comparisons with ‘control’ populations. Studies measuring at least one of eight high-risk sexual practices either as a primary or secondary outcome were included. High-risk sexual practices included early sexual debut, unprotected sex (inconsistent condom use/ contraception use), having an older partner, transactional sex, having multiple sexual partners, sex whilst intoxicated, sexually transmitted infections, and unwanted adolescent pregnancies, or a composite measure of two of these outcomes–as defined by each study. Reports in English and French were reviewed to allow for publications from Western and Central Africa.

**Exclusion criteria:** Studies of special populations such as sex workers, men who have sex with men, truck drivers, male factory workers, were excluded for three main reasons. First, the focus of the review was adolescents and youth living with HIV in Sub-Saharan Africa, not key populations. Second, these key populations at high risk of HIV-infection are likely to report high rates of sexual risk-taking which follow patterns not similar to those among adolescents living in HIV-endemic communities, and thus may have biased any conclusions reached by this review. Third, the majority of the studies of key populations focused on HIV-negative populations including only small sub-samples of HIV-positive participants.

**Search Strategy**: In September-November 2015, the first author searched the online databases of PsycARTICLES, Embase, Global Health, MEDLINE, and PsycINFO, PubMed, CINAHL, ProQuest, and WHO Afro Library, the Cochrane and Campbell databases and the PROSPERO register of systematic reviews. The first author also searched International AIDS Society conference abstracts and presentations, as well as websites of major international and regional organisations, such as the World Health Organization (WHO), Joint UN Program for HIV/AIDS (UNAIDS), the UN Children’s Fund (UNICEF), United States Agency for International Development (USAID), UN Family Planning Agency (UNFPA), International Planned Parenthood Federation (IPPF), and Population Council. Key search terms for sample population (children, adolescents, teenagers and youth), all high-risk sexual practices, location (sub-Saharan Africa) and timeline were included (S2–S4 Tables). All searches were conducted within the publication date limits of 1983 or the closest date limit available, reflecting the time since HIV has been diagnosed in adolescents and youth. Search terms were adapted to include the requirements of different databases and were included in the systematic review protocol: (PROSPERO registration number CRD42015025871).

**Screening:** The screening process followed the Cochrane Collaboration Handbook guidelines [45]. Following merging and de-duplication, two authors reviewed titles and abstracts for relevance. When available, full-text documents were retrieved and checked for eligibility against inclusion and exclusion criteria (S1 Table), and a set of pre-agreed screening questions (S5 Table). Email requests for clarifications, unpublished data, and data from published studies were sent to researchers working on sexual risk-taking of HIV-positive adolescents and youth. Recent guidance on systematic reviews suggests that there is a potential bias from including studies with very small samples in systematic reviews [46]. To minimise this bias, when studies reported ≥50 HIV-positive adolescents and youth, but age-disaggregated data was not available in the included reports, authors were contacted for age-disaggregated data for 10–24 year old HIV-positive participants. If additional data were provided, the studies were included in the review. Reference lists of the included studies and of other relevant reviews were screened for further eligible titles.

**Data extraction:** Data was extracted from full-text records by the first author (ET) using a pre-piloted data extraction form (S1 File). A second independent reviewer checked the data extraction for each included study (MP/FM/RH) and any discrepancies were resolved through discussion. Records reporting analyses from the same dataset were checked for data duplication, with the largest sample taken if multiple reports were available for the same outcome measure. For longitudinal studies reporting a change in an outcome of interest, baseline values of the reported outcome were extracted as prevalence. If data was not reported for HIV-positive adolescents or youth specifically but authors provided the raw data, the prevalence for sexual risk-taking was calculated for HIV-positive adolescent or youth, via frequencies in SPSS. In such instances, the same definition of the sexual risk outcome as the primary study was used. For example, Viegas and colleagues reported rates of early sexual debut defined as ‘before the age of 18’ for a sero-assorted sample [47]. Using a dataset shared by the research team, this review’s first author computed the prevalence of ‘sexual debut before 18 years old’ for HIV-positive youth. Where relevant, the prevalence of risky sexual practice was computed based on the prevalence of related safe sexual practices reported. For example, if a study reported condom use at last intercourse as 40%, the rate of unprotected sex at last intercourse was computed as 60%. Both reported and computed prevalence of inconsistent condom use/ unprotected sex are reported.

**Risk of bias** across studies was assessed using a Study Quality Checklist and risk assessment form (S2 File). The form drew from guidance on assessing systematic bias from the Cochrane Handbook for experimental designs (randomised controlled trials (RCTs), non-randomised controlled trials and pre- and post-test experimental design) [45], and the Cambridge Quality Checklist for systematic reviews of risk factors [48]. The checklist was adapted in line with a systematic review of internalised stigma among people living with HIV [49]. Adaptation included assessing sampling strategies at two levels: facility/ community and individual level, and assessing each individual association between potential factors and the outcome of interest. For each potential determinant, each outcome-determinant relationship was scored as a percentage of the total score possible from the Study Quality Checklist (SQC). SQC scores for each outcome-predictor relationship are reported in S6 Table.

**Data synthesis:** Given the diversity of primary studies and outcomes measured, and the cross-sectional nature of the majority of the included studies, a meta-analysis was not conducted, in order to avoid potentially misleading conclusions [48]. To reflect the diversity of reported prevalence rates, data was reported as the range of reported values for studies using the same definitions for each outcome.

## Results

The results of this review are reported in five sections: (1) characteristics of included studies, (2) quality assessment of included studies, (3) prevalence of sexual risk outcomes, (4) factors associated with sexual risk-taking, and (5) interventions addressing sexual risk-taking.

### Characteristics of included studies

**Fig 1:**
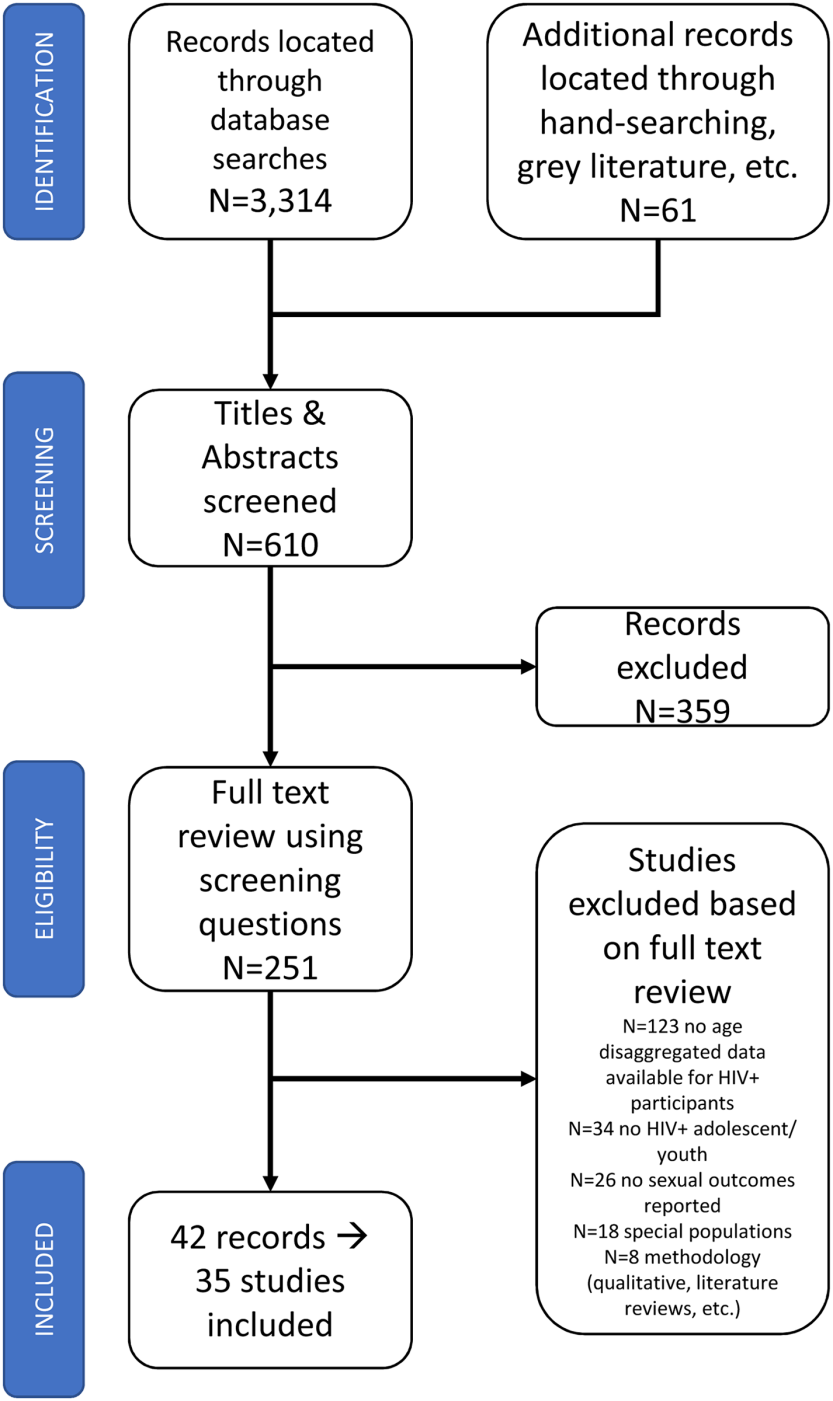
PRISMA flow diagram for identifying included studies.

Fig 1 shows the PRISMA flow diagram for included studies. The PRISMA checklist is available as supporting information. Results from different database searches were merged, resulting in 3,314 records. Grey literature searches and hand-searches of references of included studies resulted in an additional 61 records. After de-duplication, two authors (ET/KK) reviewed 610 titles and abstracts and the full text documents for 251 results. A total of 42 records were included in this systematic review, which reported data from k = 35 studies (Table 2).

**Table 2:** Summary of included studies.

*First author*, *year*	*Location*	*Study design*^1^	*Sampling strategy*^2^	*Target population*	*HIV+ adolescents/ youth*
Ankunda 2011 [77]; Ankunda 2016 [50]	Uganda	CS	FP; TR	15–24 year old HIV+	n = 425 (2011) n = 335 (2016)
Bakeera-Kitaka 2008 [58]	Uganda	CS	FP; PS	15–24 year old HIV+	n = 75
Banura 2008 [57]	Uganda	CS	FP; CS	12–24 year old HIV+ and HIV-	n = 82
Baryamutuma 2010 [59]	Uganda	CS	FP; CS	13–19 year old HIV+	n = 386
Beyeza-Kasheysa 2011 [60]	Uganda	CS (PCS)	FP; CS	15–24 year old HIV+ & HIV-	n = 276 (2009) n = 206 (2011)
Birungi 2009 [51]; Birungi 2009 [74]; Obare 2010 [118]	Uganda	CS	FP-CP; CS	15–19 year old HIV+ and status unknown)	n = 732
Birungi 2011 [61]	Kenya	CS	FP; PS	15–19 year old HIV+ females	n = 757
Cataldo 2012 [28]	Malawi, Mozambique, Zambia, Zimbabwe	CS	NR; CS	10–19 year old HIV+	n = 1,703
Gavin 2006 [68]	Zimbabwe	CS	CRS; TR	15–19 year old	n = 192
Gray 1998 [69]	Uganda	CS (RCT)	CRS; TR	15–49 year old females	n = 361
Heffron 2010 [70]	Eastern Africa: Kenya, Rwanda, Tanzania, Uganda Southern Africa: Botswana, South Africa, Zambia	CS	CRS; TR	18–45 year old females	n = 523
Hendriksen 2007 [71]; Steffenson 2011 [83]	South Africa	CS	CRS; TR	15–24 year old	n = 1,235 (2007); n = 1,091 (2011)
Hoffman 2008 [62]	Malawi	CS (PCS)	FP; PS	18+ year old HIV+ females	n = 90
Holub 2010 [63]	Democratic Republic of Congo	CS	FP; PS	14–24 year old HIV+	n = 103
Kaggwa 2012 [125]	Uganda	CS	FP; NR	16–24 year old HIV+	n = 453
Katusiime 2012 [56]	Uganda	CS	FP; PS	15–24 year old HIV+	n = 148
Kembo 2012 [72]	Zimbabwe	CS	CRS; NR	15–24 year old HIV+ and HIV-	n = 477
Lightfoot 2007 [41]	Uganda	RCT	CNR; CS	14–21 year old HIV+	n = 100
Malaju 2013 [67]	Ethiopia	CS	FP; NR	15–24 year old HIV+ and HIV-	n = 104
Mbalinda 2015 [52]; Mbalinda 2015 [114]	Uganda	CS	FP-FRS; PS	10–19 year old HIV+	n = 624
Mhalu 2013 [76]	Tanzania	CS	FRS; TR	15–24 year old HIV+	n = 232
Morris 2012 [31]	Cameroon	CS	FRS-CP; TR	12–26 year old HIV+, HIV-, status unknown	n = 114
Muyindike 2012 [65]	Uganda	CS	FP; NR	18–49 year old women (n = 826)	n = 211
Nhamo 2013 [87]; Nhamo 2014 [78]	Zimbabwe	RCT	NR; NR	16–19 year old HIV+	n = 710
Nöstlinger 2015 [55]	Uganda	CS (PCS)	FC-CC; PS	13–17 year old HIV+	N = 532
Obare 2010 [53]	Kenya	CS	FP; PS	15–19 year old HIV+	n = 606
Pascoe 2015 [73]	Zimbabwe	CS	CRS; TR	18–22 year old HIV+ and HIV-	n = 199
Santelli 2013 [32]	Uganda	CS (PCS)	CRS; TR	15–24 year old HIV+ and HIV-	n = 204
Senyonyi 2012 [42]	Uganda	RCT	FP; CS	12–18 year old HIV+	n = 115
Shisana 2014 [29]	South African	CS	CRS; TR	All ages HIV+ and HIV-	n = 443
Snyder 2014 [40]	South Africa	PPT	FC-CC; CS	16–24 year old HIV+	n = 65
Test 2012 [75]	Rwanda	CS	FC-CC; CS	16–24 year old HIV+	n = 107
Toska 2015 [54]	South Africa	CS	FRS-CP; TR	10–19 year old HIV+	n = 858
Viegas 2015 [47]	Mozambique	CS	FC; PS	18–24 year old HIV+ and HIV-	n = 85
Wanyenze 2011 [66]	Uganda	CS	FP; CS	15–49 year old HIV+	n = 159

^1^ CS cross-sectional study, PCS prospective cohort survey, PPT pre and post-test; RCT randomised controlled trial.

^2^ Sampling strategy documented at two levels: (1) cluster: facility random and/ or stratified (FRS); facility purposeful (FP), community random and/or stratified (CRS), community purposeful (CP), mixed–see symbols (2) individual: total/ random (TR), purposeful (PS), convenience (CS); NR–not reported.

#### Study design

The 35 included studies reported data from N = 13,536 HIV-positive adolescents and youth living in 13 countries. Four studies described interventions evaluated through RCTs (k = 3) or pre- and post-test experimental design (k = 1), and the remaining k = 31 reported on cross-sectional data.

#### Participant characteristics

Participants were mostly female (k = 35 studies: 47%-100%), vertically infected (k = 9 studies: 43%-100%), and on ART (k = 9 studies: 0%-88%). Of the 17 studies that reported whether HIV-positive adolescents and youth knew their status, the majority recruited only adolescents who knew their status (k = 13). In the six studies which recorded disclosure of HIV status to others, just under half of HIV-positive adolescents had shared their HIV-positive status with their partners (31%-74%) [50–55].

#### Outcome measures

Thirty-three studies assessed sexual practices of HIV-positive adolescents as the primary outcome, and two reported them as secondary outcomes. The outcomes measured varied in terms of the recall periods of measurement and exact definitions (Table 3). Nine studies reported on only one sexual practice, while the rest reported on two or more sexual practices. The most common definitions for each reported outcome were: (1) sex before 15 years old (k = 3), (2) sex before 18 years old (k = 3), (3) inconsistent/no condom use at last sexual encounter (k = 14), (4) current use of modern contraception (k = 6), (5) having an older partner at first sexual intercourse (k = 4), (6) having ever had transactional sex (k = 3), (7) multiple sexual partners in the past 12 months (k = 5), (8) sex while intoxicated (k = 2), (9) ever having had an STI or STI symptoms (k = 5), and (10) ever been pregnant (k = 7). All outcomes were based on self-reports, except for two studies reporting results of STI tests for Hepatitis B (HBV) [56] and Human Papillomavirus (HPV) [57]. One study reported on a composite sexual risk-taking score [42]. In addition to the above high-risk sexual practices, three studies reported on risk-exposure sexual outcomes such as forced sex, non-consensual first sex, gender-based violence, as their main outcomes. These outcomes were beyond the scope of the initial study protocol, therefore information on sexual risk-exposure outcomes is provided in S7 Table.

**Table 3:** Prevalence rates of sexual risk-taking among HIV-positive adolescents and youth.

*Outcome*	*Definition*	*Studies*	*Rates (% or M)*	*Gender or age disaggregation* *(NR–not reported)*	*Actual reported outcome (actual value reported in the study)*
Early sexual debut	Not defined	Ankunda 2011 [77]	35.1%	NR	Abstinence/ never having had sex (64.9%
		Baryamutuma 2010 [59]	39.9%	NR	Early sexual initiation
		Birungi 2009 [51]	33%	31% F; 37%M	Ever had sex (all under 19 years old)
		Cataldo 2012 [28]	Mal: 10%; 38%	NR	Ever had sex (all under 19 years old)
			Moz: 11%; 65%	Moz: 10–14 y.o. (4%F; 17% M) 15–19 y.o. (69% F; 49% M)	
			Zam: 5%; 29%	NR	
			Zim: 3%; 16%	NR	
		Lightfoot 2007 [41]	40%	NR	Ever had sex (all under 19 years old)
		Obare 2010 [118]	84%	88% F; 73% M	Ever had sex (all under 19 years old)
		Toska 2015 [54]	15.1%	19.2% F; 10.7% M	Ever had sex (all under 19 years old)
	Age of sexual debut	Ankunda 2016 [50]	16.9 (2.62)	17.1 (2.41) F; 15.4 (3.85) M	Mean age (SD) of sexual debut
		Gavin 2006 [68]	16.4 (0.14)	NR	Mean age (SD) of sexual debut
		Morris 2012 [31]	17.2 (2.3)	NR	Mean age (SD) of sexual debut
		Nöstlinger 2015 [55]	13 (11–14)	NR	Median age (IQR) of sexual debut
		Test 2012 [75]	17 (15–18)	17 (15–18) F; 16 (15–17.5) M	Median age (IQR) at consensual sexual debut
	<15 years old	Kembo 2012 [72]	28.1%	NR	
		Mhalu 2013 [76]	51.5%	68% F; 84.8% M	
		Shisana 2014 [29]	7.4%	4.4% F; 19.1% M	
	≤15 years old	Bakeera-Kitaka 2008 [58]	42.1%	NR	
		Holub 2010 [63]	48.5%	45% F; 65% M	
	<18 years old	Malaju 2013 [67]	63.6%	NR	
		Pascoe 2015 [73]	36.2%	NR	
		Viegas 2011 [47]	77.6%	78.6% F; 70% M	
	≤19 years old	Kembo 2012 [72]	87.0%	NR	Sex between 15–19 58.9% & sex before 15 28.1%
Inconsistent condom use/Unprotected sex	Not clearly defined	Ankunda 2011 [77]	61.7%	NR	Condom use (38.3%)
		Baryamutuma 2010 [59]	56.3%	NR	Consistent condom use (43.7%)
		Beyeza-Kashesya 2011 [60]	28.2%	NR	No contraception at all (28.2%)
		Heffron 2010 [70]	76%	NR	Consistent condom use (24%)
		Mhalu 2013 [76]	58.6%	NR	Unprotected sex
		Pascoe 2015 [73]	58.6%	NR	Used condoms at every sexual act (41.4%)—timeline not defined
			82.4%	NR	Sometimes/ never used condoms with any partners (82.4%)
	Never	Beyeza-Kashesya 2011 [60]	39%	NR	Never used condoms (39%)
		Morris 2012 [31]	11.9%	NR	Ever used condoms (88.12%)
		Nöstlinger 2015 [55]	43.5%	NR	Ever used a condom (56.5%)
	First sex	Kembo 2012 [72]	77.4%	NR	Condom use (22.6%)
		Nöstlinger 2015 [55]	75%	NR	Condom use at first sex (25%)
		Toska 2015 [54]	23.6%	21.1% F;; 16.2% M	Unprotected sex (25.8%, 23.6% F, 16.2% M)
	Last sex	Ankunda 2016 [50]	46.7%	NR	Consistent condom use (53.3%)
		Gavin 2006 [68]	80.3%	All female	Condom use at last sex (19.7%)
		Hendriksen 2007 [71]	37.9%	51.3% F; 55% M	Unprotected sex
		Holub 2010 [63]	18.5%	NR	Unprotected sex
		Kaggwa 2012 [125]	49.5%	NR	No condom use at last sex (49.5%)
		Kembo 2012 [72]	82.7%	NR	Condom use (17.3%)
		Mbalinda 2015 [114]	43.7%	NR	Unprotected sex (43.7%)
		Mhalu 2013 [76]	55.3%	NR	Condom use (44.7%)
		Morris 2012 [31]	45%	NR	Condom use (55%)
		Pascoe 2015 [73]	58.8%	NR	Unprotected sex
		Shisana 2014 [29]	39.1%	NR	Condom use at last sex (60.9%, 62.2% F, 55.5% M)
		Snyder 2014 [40]	29%	37.8% F; 44.5% M	Condom use at baseline (pre-intervention 71%)
		Toska 2015 [54]	28.9%	31.9% F; 13.5% M	Unprotected sex at last sex (28.95%, 31.9% F, 13.5% M)
		Viegas 2015 [47]	37.6%	38.7% F; 30% M	Condom use (62.4%, 61.3% F, 70% M)
	Last 3 months	Lightfoot 2007 [41]	87.5%	NR	Always use condoms at baseline for full intervention sample (12.5%)
	Last 6 months	Ankunda 2016 [50]	53.6%	NR	Consistent condom use (46.4%)
		Mbalinda 2015 [52]	77.5%	NR	Use at every sexual act (22.5%)
		Test 2012 [75]	56%	57% F; 50% M	Inconsistent condom use (56%, 57% F, 50% M)
	Last 12 months	Santelli 2013 [32]	88.7%	92.6% F; 78.6% M	Never or inconsistent condom use (88.7%, 92.6% F, 78.6% M)
	Current	Birungi 2009 [51]	48%	41% F; 52% M	Condom use: sometimes or rarely
		Obare 2010 [118]	60.7%	61.8% F; 59.2% M	Current using condoms (39.3%, 38.2% F, 40.8% M)
		Morris 2012 [31]	69.3%	NR	Condom use: sometimes or never (69.3%) vs. always (30.7%)
		Obare 2010 [118]	14%	16% F; 4% M	Condom use (86%, 84% F, 96% M)
Contraception	Ever any method	Beyeza-Kasheysa 2009 [60]	33%	33%	Ever used any contraception (n = 345)
		Birungi 2009 [74]	49.6%	43.8% F; 58.2% M	Any contraception used in past/current relationships
		Obare 2010 [118]	72%	72% F; 70% M	Ever used contraception
		Wanyenze 2011 [66]	78.7%	NR	Any family planning method (including traditional)
	Ever pill/ injectable	Obare 2010 [118]	12.5%	All female	Pill OR injectable among sexually active females
	First sex	Birungi 2009 [51]	36.4%	37.5% F; 34.7% M	Used a method to prevent HIV infection/ reinfection at first sex
		Obare 2010 [118]	14%	15% F; 12% M	Any method
	Current modern	Beyeza-Kashesya 2011 [60]	33.9%	NR	Contraception use at baseline
		Obare 2010 [118]	42.6%	41% F; 44.9% M	Current modern contraception
		Hoffman 2008 [62]	36.7%	All female	At baseline
		Muyindike 2012 [65]	29.9%	All female	Current use at enrolment in study
		Obare 2010 [118]	66%	65% F; 68% M	Any method
		Wanyenze 2011 [66]	63.8%	NR	Any modern method of family planning
		Wanyenze 2011 [66]	25.5%	NR	Any effective method of family planning (excluding condoms)
	Current pill/ injectable	Beyeza-Kashesya 2011 [60]	21%	All female	Hormonal contraception
	Oral contraceptives	Heffron 2010 [70]	4.0%	All female	At baseline
		Obare 2010 [118]	6%	6% F; 3% M	At baseline
	Injectable	Heffron 2010 [70]	14.7%	All female	At baseline
		Obare 2010 [118]	23%	28% F; 2% M	At baseline
	Implants	Heffron 2010 [70]	0.6%	All female	At baseline
		Obare 2010 [118]	2%	2% F; 2% M	At baseline
	Hysterectomy	Heffron 2010 [70]	0.6%	All female	At baseline
	Post-partum contraception	Birungi 2011 [61]	61%	All female	After pregnancies had ended
	Contraception uptake	Beyeza-Kashesya 2011 [60]	28.4%	NR	Started using any methods of contraception in past 12 months
	Long-term contraception use	Beyeza-Kashesya 2011 [60]	17.8%	NR	At baseline, 6 and 12 months follow up
		Heffron 2010 [70]	26.3%	All female	% quarterly visits reporting contraception use during 24-month follow-up
	Discontinued contraception	Beyeza-Kashesya 2011 [60]	23.6%	NR	Stopped using contraception during 12 month follow-up period
	Dual method use	Beyeza-Kashesya 2009 [60]	5%	NR	Unclear definition
Older sexual partner	First sexual partner	Gavin 2006 [68]	6.6 (0.87)	All female	Age difference to partner–mean (SD)
		Morris 2012 [31]	65.7%	NR	6+ years older
		Obare 2010 [118]	52%	61% F; 20% M	Older
		Obare 2010 [118]	4%	4% F; 3% M	Much older
		Test 2012 [75]	66%	75% M; 17% M	>5 years older
		Test 2012 [75]	37%	41% F; 17% M	>10 years older
	Any	Pascoe 2015 [73]	28.1%	NR	6–10 years old
		Pascoe 2015 [73]	18.1%	NR	11+ years older
	Last sexual partner	Gavin 2006 [68]	5.0 (0.67)	All female	Age difference between participant and partner–mean (SD)
	Current sexual partner	Obare 2010 [118]	56%	68% F; 10% M	Older partner
		Obare 2010 [118]	9%	11% F; 1% M	Much older
		Shisana 2014 [29]	35.4%	40.6% F; 12.7% M	Other options: 5+ years younger, less than 5 years difference
Transactional sex	Ever	Holub 2010 [63]	22.3%	23.3% F; 17.6% M	Ever received money for sex
		Pascoe 2015 [73]	22.6%	NR	Had sex with partner for material/other support
		Test 2012 [75]	20.6%	66% F; 17% M	Ever had sex for money
	Last partner	Gavin 2006 [68]	37.9%	NR	Received goods or money for sex with the last partner
	Not clearly defined	Nhamo 2014 [78]	60%	All female	Not clear, at baseline of RCT
Multiple sexual partners	Number of lifetime partners	Beyeza-Kashesya 2011 [60]	3	NR	Unclear if mean or median, IQR (1–4)
		Test 2012 [75]	2.5 (1–5)	3 (1–6) F; 3 (2–4.8) M	Median (IQR)
	Lifetime	Gavin 2006 [68]	15.7%	All female	Multiple lifetime sexual partners
		Morris 2012 [31]	81.2%	NR	2 or more lifetime sexual partners
		Pascoe 2015 [73]	26.1%	NR	2 or more lifetime sexual partners
		Viegas 2015 [47]	88.2%	86.7% F; 100% M	More than 1 lifetime sexual partner
	Last 6 months	Mbalinda 2015 [114]	16%	NR	More than 1 partner
		Nhamo 2014 [78]	6%	All female	Multiple sexual partners at baseline of RCT
	Last 12 months	Steffenson 2011 [83]	11.5%	7.1% F; 30% M	Concurrent partnerships
		Holub 2010 [63]	7.7%	9% F; 0% M	2 or more partners
		Kembo 2012 [72]	4.6%	NR	2 or more sexual partners
		Santelli 2013 [32]	18.1%	12.2% F 33.9% M	2 sexual partners
		Santelli 2013 [32]	10.8%	2.0% F; 33.9% M	3 or more sexual partners
		Santelli 2013 [32]	9.8%	2.0% F; 30.4% M	2 or more sexual partners from outside the community
		Shisana 2014 [29]	15.4%	11.8% F; 29.5% M	2 or more sexual partners
	Current	Ankunda 2016 [50]	30.4%	NR	At time of interview
		Beyeza-Kashesya 2011 [60]	18%	NR	Current polygamous relationships
		Mhalu 2013 [76]	14.5%	15.9% F; 10.6% M	Compared to those with 0–1 sexual partners
		Santelli 2013 [32]	13.2%	4.7% F; 35.7% M	Concurrent partnerships at interview
	Not clearly defined	Bakeera-Kitaka 2008 [58]	36.8%	NR	2 or more sexual partners
Sex intoxicated	Sex after alcohol	Shisana 2014 [29]	5.5%	5.8% F; 4.2% M	Drank alcohol before sex with most recent partner
		Shisana 2014 [29]	3.7%	4.9% F; 1.7% M	Drank alcohol before sex with the second most recent partner
		Test 2012 [75]	29%	NR	Drank alcohol up to 6 hours before sex
STIs	Ever had STIs	Baryamutuma 2010 [59]	17.6%	NR	Self-reported
		Gavin 2006 [68]	21.9%	All female	Self-reported symptoms
		Mbalinda 2015 [52]	13.1%	NR	Ever had STI treatment (self-reported)
		Mbalinda 2015 [52]	14.8%	NR	Ever had STI symptoms: genital sores
		Mbalinda 2015 [52]	27.7%	NR	Ever had STI symptoms: genital itching
		Mbalinda 2015 [52]	10.9%	NR	Ever had STI symptoms: genital discharge
		Mbalinda 2015 [52]	16.8%	NR	Ever had STI symptoms: lower abdominal pain
		Pascoe 2015 [73]	46.2%	NR	Ever had STI symptoms
		Viegas 2015 [47]	36.5%	34.7% F; 50% M	Self-reported
	Last 6 months	Toska 2015 [54]	13.8%	NR	2+ STI symptoms
	Last 12 months	Kembo 2012 [72]	5.4%	NR	Self-reported occurrence
		Santelli 2013 [32]	25.5%	20.9% F; 37.5% M	STI symptoms: genital ulcers
		Santelli 2013 [32]	35.8%	42.6% F; 17.9% M	STI symptoms: genital discharge
		Santelli 2013 [32]	39.2%	All female	STI symptoms: vaginal discharge
		Santelli 2013 [32]	64.2%	All female	STI symptoms: vaginal itching
		Santelli 2013 [32]	16.2%	All female	STI symptoms: unpleasant vaginal odour
		Santelli 2013 [32]	14.7%	16.9% F; 8.9% M	STI symptoms: frequent urination
		Santelli 2013 [32]	23.0%	22.3% F; 25% M	STI symptoms: painful urination
		Santelli 2013 [32]	11.3%	12.8% F; 7.1% M	STI symptoms: pain during intercourse
		Santelli 2013 [32]	1.5%	2.0% F; 0% M	STI symptoms: bleeding during intercourse
		Santelli 2013 [32]	29.4%	35.8% F; 12.5% M	STI symptoms: lower abdominal pain
		Santelli 2013 [32]	6.9%	8.1% F; 3.6% M	STI symptoms: genital warts
	Current STI	Banura 2008 [57]	87.8%	NR	HPV–any infection (single or multiple strain–biomarker)
		Banura 2008 [57]	64.6%	NR	HPV–single strain (biomarker)
		Banura 2008 [57]	23.2%	NR	HPV–multiple strains (biomarker)
		Katusiime 2012 [56]	6.1%	NR	HBV–positive for HBsAg (biomarker)
		Pascoe 2015 [73]	49.7%	NR	HSV-2 infection (biomarker)
Pregnancy	Ever	Baryamutuma 2010 [59]	13.2%	NR	Ever pregnant/ impregnated someone
		Obare 2010 [118]	41%	All female	Ever pregnant among sexually active females
		Birungi 2011 [61]	52%	All female	Ever pregnant
		Gavin 2006 [68]	15%	All female	Ever pregnant: 13.7% among 10–14 y.o.; 20.6% among 15–19 y.o.
		Mbalinda 2015 [52]	49%	56.9% F; 33.3% M	Ever been or made someone pregnant
		Nhamo 2014 [78]	40%	All female	At baseline of RCT
		Obare 2010 [118]	60%	68% F; 27% M	Ever been or made someone pregnant
	Current	Beyeza-Kashesya 2011 [60]	5%	NR	Pregnant at the time of the study
		Gray 1998 [69]	15%	All female	20.6% in 10–15 year olds; 13.7% in 20–24 year olds
	Multiple pregnancies	Birungi 2011 [61]	24.1%	All female	
	Unintended pregnancy	Birungi 2011 [61]	73.9%	All female	Among those reporting at least one pregnancy
		Nhamo 2014 [78]	75%	All female	Baseline of RCT, among all pregnancies
		Obare 2010 [118]	75%	NR	
Safe sex	Not reported	Cataldo 2012 [28]	Malawi: 29%; 53%; Mozambique: 20%; 73%; Zambia: 33%; 70%; Zimbabwe: 10%-37%	NR	Among sexually active participants only (rate among 10–14 year olds; rate among 15–19 year olds)
	Highly protected sex	Lightfoot 2007 [41]	69.5%	NR	Abstinent or consistent condom use at RCT baseline
Composite sexual risk-taking	HIV transmission risk behaviour	Senyonyi 2012 [42]	2.11 (SD 2.75) intervention group; 1.83 (SD 2.57) control group	NR	Score of several behaviours: number of sexual encounters (intercourse or penetrative sex) + number of sexual partners + unprotected penile penetrative vaginal sexual acts

### Quality assessment of included studies

Of the seventeen studies that reported on potential risk factors or intervention effects, most scored below 50% in the Study Quality Checklist (k = 14, range 10%-75%, S6 Table). The reasons for the low scores included study design and analyses, sampling strategies, response/retention rates, and sample size, which are discussed in this section.

#### Study design

Of the included studies sixteen were cross-sectional, three were prospective cohorts, one was an experimental pre- and post-test, and seven were RCTs. The three prospective cohort studies did not report analyses of change, nor did they assess longitudinal predictors of sexual risk-taking; hence, only relevant baseline data was extracted on prevalence and potential associated factors. Data from four RCTs reported only on prevalence or factors associated with the outcomes of interest using cross-sectional data from the baseline of the study. The included data on prevalence and potential factors associated with sexual risk-taking were cross-sectional (k = 31), while intervention data was based on a pre- and post-test experimental study (k = 1) and three RCTs (k = 3).

#### Sampling

Sampling strategies were assessed at two levels: community/clinics and individual level (Table 2). Of the 31 observational studies, seventeen recruited only from healthcare facilities, primarily through purposefully selected facilities (k = 14) [50,53,56–67]. Most of these studies recruited only HIV-positive participants (k = 11). Seven other studies recruited only from communities through random or stratified sampling [29,68–73]. Most of these studies recruited HIV-positive participants as part of larger community-based samples. Five studies recruited participants through combined facility/community sampling [31,54,55,74,75].

At the individual-level sampling, eleven studies recruited through total or random sampling at each study site [29,31,32,50,54,68–71,73,76], eight recruited through purposeful sampling [47,52,55,56,58,61–63], and another eight through convenience sampling [28,53,57,59,60,66,74,75]. The three intervention studies which reported sampling data recruited through a combination of purposeful and convenience sampling at both the facility/community and individual levels [40–42].

#### Sample sizes

The included studies reported on n = 13,536 HIV-positive adolescents and youth (10–24 years old), with sample sizes ranging between n = 65 and n = 1,703. Fourteen of thirty-five studies had a sample size smaller than n<400 participants, which was chosen as the cut-off for a study powered to detect predictors and intervention effects, based on a recent systematic review which assessed studies of predictors of internalised HIV-stigma [49]. Studies with sample sizes <400 scored lower in the Study Quality Checklist.

#### Response and retention rate

Most studies (k = 26) did not report response or retention rates or did not have response rates for the HIV-positive sub-population, making it difficult to assess the extent of selection bias. Of studies that reported response or retention rates (k = 9), the majority stated retention of ≥90% [41,42,53,54,60,74,75,77], with only one reporting a retention rate of 89.6% [28]. Two of the three small-scale intervention studies analysed only data from completers, who accounted for 67.3% [42] and 59.6% [40] of those who enrolled in the studies.

#### Strength of associations between factors/ interventions and outcomes

In the cross-sectional data analyses, 13 studies conducted univariate analyses (such as Chi square tests, univariate logistic regressions, or Student’s t-tests) and eleven reported on multivariate analyses (such as logistic regressions, multivariate log binomial regression, random effects logit model estimations) of associations between potential factors and the outcomes, controlling for potential confounders (S6 Table). All four experimental design studies reported within-group change for at least one sexual behaviour over time, with three randomising participants to a control and an intervention group [41,42,78].

### Prevalence of sexual risk-taking (Table 3, S6 Table)

Prevalence of high-risk sexual practices varied widely by outcome and should be interpreted with caution alongside data on study methodology, sampling strategy and size, response rates, and definitions of outcome measures. Nonetheless, several trends were notable. First, most studies reported high prevalence of sexual risk-taking in relatively young populations (<19 years old). The prevalence of inconsistent condom use varied considerably, though most of the studies found that between one-third and half of participants reported unprotected sex at last intercourse. These prevalence rates are comparable to those reported by studies in the general adolescent population in South Africa [71], Uganda [32], and other sub-Saharan African countries [79,80]. Of the studies reporting current contraception use, one-third of the sample were on a form of contraception at the time of the study, but only one study documented extremely low prevalence of dual protection– 5% of combined contraception and condom use–in Uganda [60]. These results suggest that a high proportion of sexual acts by HIV-positive adolescents and youth in sub-Saharan Africa are unprotected.

Second, where studies reported sexual debut, nearly half of the adolescents had had sex. This is an important finding since most of the included participants were HIV-positive adolescents under 19 years old, which suggests early sexual debut for a large part of the HIV-positive adolescent population. Early sexual debut has been linked to reduced protected sex in the general adolescent population in South Africa [81]. Third, between half and two-thirds of adolescents reported having an older sexual partner during first sex, which is linked to reduced condom use in the general adolescent population [68]. Fourth, one in five participants reported engaging in transactional sex or having sex for money or goods–a sexual practice associated with unprotected sex [82], a result that was consistent across multiple studies [63,73,75]. HIV-positive adolescent girls reported a higher prevalence of transactional sex, having older sexual partners, and unprotected sex, though adolescent boys were more likely to report early sexual activity and multiple sexual partners.

Fifth, the prevalence of multiple sexual partners varied widely, though most studies reported that at least one in ten participants had had at least two sexual partners in the last 12 months and one in three male participants reported multiple sexual partners [29,32,83]. Sixth, studies testing for biomarkers of HSV-2 and HPV found 50% and 88% infection rates in HIV-positive adolescents, respectively [57,73]. This suggests high levels of unprotected sex and higher risk for co-infection with other sexually transmitted infections.

### Factors associated with sexual risk-taking among HIV-positive adolescents and youth (Table 4)

**Table 4:** Factors associated with sexual-risk taking reported in included studies.

*Factor level/ grouping*	*Factor*	*Increased sexual risk-taking*	*Non-significant associations*	*Decreased sexual risk-taking*
Individual–Socio-demographic factors	Age (older)	Ever had sex [52]	Condom use [64], unprotected sex [54], multiple sexual partners [64,76]	Unprotected sex [76], condom use [64,77], contraception use^1^ [51]
	Gender (female)		Unprotected sex [54,76], contraception use [51], multiple sexual partners[76]	
	Rural residence		Unprotected sex [54]	
	Informal housing		Unprotected sex [54]	
	Study site		Contraception use [61], unintended pregnancy [61], unprotected sex [76], multiple sexual partners [76]	
Individual–Mental and physical health factors	Depression (clinical)		Multiple sexual partners [64]	Condom use [64]
	Anxiety		Condom use [64], multiple sexual partners [64]	
	Poor birth outcomes		Contraception use [61]	
Individual–Knowledge, attitude, and beliefs	Does not drink alcohol		Multiple sexual partners [76]	Ever had sex [52], unprotected sex [76]
	STI prevention knowledge		Unprotected sex [76], multiple sexual partners [76]	
Relationship-level factors	Has children with husband	Unintended pregnancy [61]	Contraception use^3^ [61]	
	Living arrangement: lives with partner	Contraception use [51], unintended pregnancy [87]		
	Gender-based violence	Unintended pregnancy [61,87], multiple sexual partners [87]		
Family and community-level factors	Lives with biological parent		Unprotected sex [54]	
	Lives alone	Ever had sex [52]		
	Orphanhood		Unprotected sex [54,63]	
	Parental monitoring*social support		Unprotected sex^2^ [63]	
	Social support		Condom use [64], multiple sexual partners [64]	
Structural-level factors	Education		Unprotected sex [76], multiple sexual partners [76]	Ever had sex [114]
	Maternal education		Contraception use^3^ [61], unintended pregnancy [61]	
	Poverty		Unprotected sex [54]	
	Food insecurity	Unintended pregnancy [87]		
	Employment	Ever had sex [52]		
	Intervention–combination social protection (grants + livelihood training + SRH services)		Multiple sexual partners [87]	Condom use [87], transactional sex [87]
HIV-related factors	Knows own HIV+ status			Unprotected sex [54]
	Mode of infection (vertical)		Condom use [64], multiple sexual partners [64]	Unprotected sex [54]
	Time since diagnosis (years)			
	Time on ART		Unprotected sex [54]	
	ART adherence		Unprotected sex [54]	
	Opportunistic infections		Unprotected sex [54]	
	Partner HIV-status unknown	Multiple sexual partners [76]	Unprotected sex [54,76]	
	Disclosed HIV status to partner		Unprotected sex [54]	
	ART use/ access		Unprotected sex [76], condom use [64], multiple sexual partners [64,76]	
	ART care (hospital vs. primary clinic)		Unprotected sex [54]	
	Intervention–access to health services: HIV support group			Condom use [40]

^1^ All contraception-related outcomes are included: at first sex, ever used any modern method, and current use of any method.

^2^ Study reported on the individual factors in univariate analyses, neither of which were significant. The interaction term was also not significant at p = 0.11.

^3^ Post-partum contraception.

**Fig 2:**
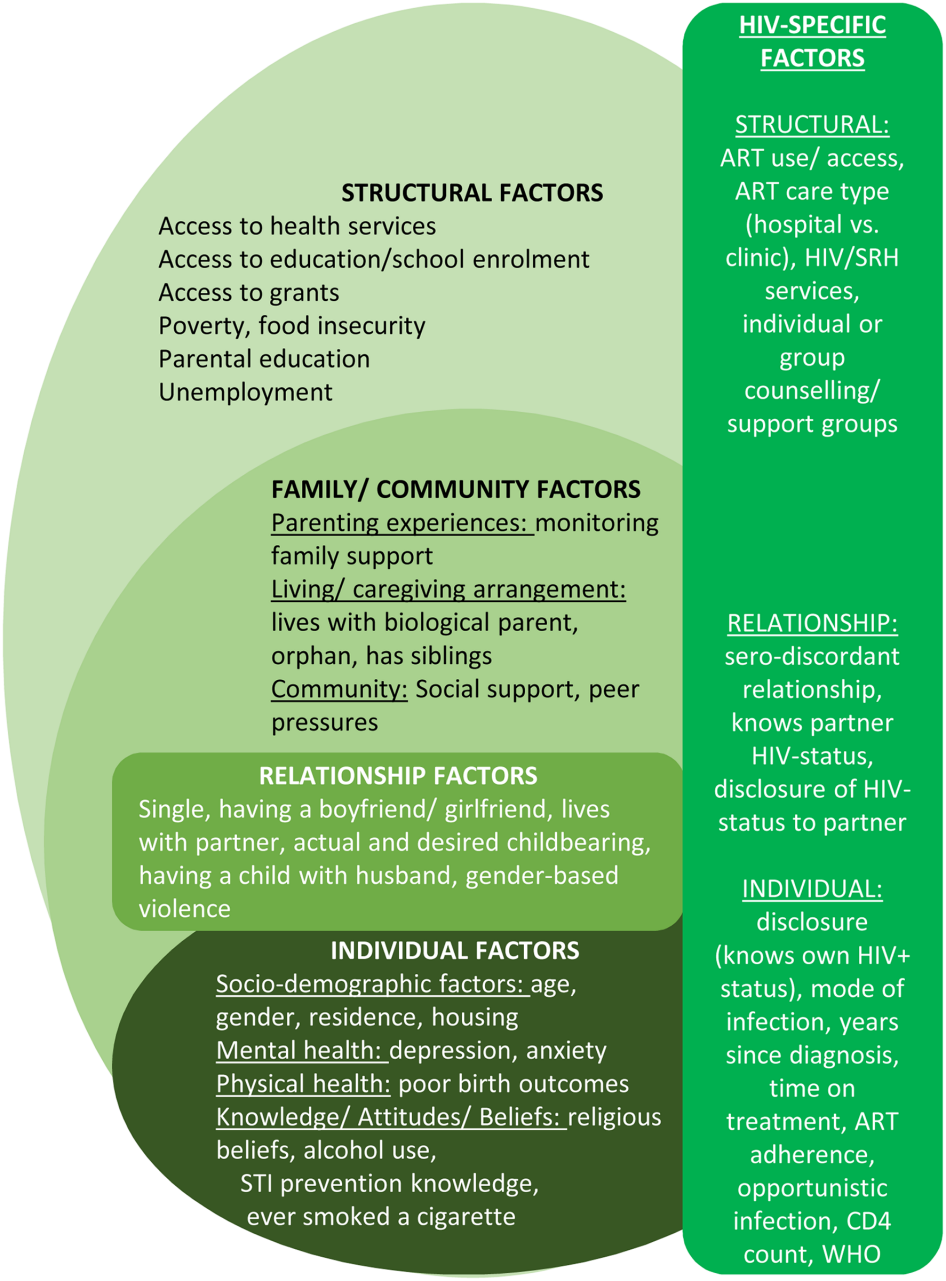
Hypothesised factors associated with sexual risk-taking among HIV-positive adolescents and youth in Sub-Saharan Africa.

Seventeen of the included studies (23 publications) reported associations between one or more factors and sexual outcomes, with univariate and multivariate analyses testing the strength of these relationships. No longitudinal predictors or causal relationships between factors and sexual risk-taking were reported by any of the included studies. Potential factors associated with sexual risk-taking among HIV-positive adolescents and youth are presented in five groups following the socio-ecological model [84–86] as a theoretical framework (Fig 2): (1) individual-level factors, (2) relationship-related factors, (3) family and community factors, (4) structural factors, and (5) HIV-specific factors. Nine studies included associations between risky sexual practices [52,54,59,61,63,75–77,87]. Most associations between different types of sexual risk-practices were not statistically significant, with. only two studies reporting significant multivariate associations using cross-sectional data: inconsistent condom use and having multiple sexual partners were associated with unintended pregnancy [61,87]. Only multivariate results are described in detail in this section with all associations and actual statistical test results–where available–included in S6 Table.

#### Individual-level factors

Seventeen studies assessed individual-level factors using multivariate analyses. *Socio-demographic* factors included: age [50,52,54,64,74,76], gender [51,54,76], rural residence [54], and informal housing [54]. *Mental and physical health factors* included: clinical depression [64], anxiety [64], and having poor birth outcomes, such as pre-mature birth, small birth weight, and child being small for gestational age [61]. *Knowledge*, *attitude*, *and behaviours* included drinking alcohol [50,76], and STI prevention knowledge [76]. Four studies reported inconsistent associations between age and sexual risk-taking: three reported that older age is associated with lower reports of high-risk sexual practices [64,74,76], but no significant associations with others [54,64,76]. Three studies reported non-significant multivariate analyses on the relationship between gender and sexual risk-taking [54,74,76], although gender-disaggregated prevalence rates suggest that HIV-positive adolescent girls/ young women engage in higher levels of risk-taking compared to HIV-positive adolescent boys/ young men [28,68,69]. Rural residence and informal housing were not significantly associated with any sexual practices.

Two studies reported mental and physical health factors associated with sexual risk-taking: depression, anxiety, and poor birth outcomes. Clinical depression was significant associated with lower condom use but not with having multiple sexual partners [64]. Anxiety and poor birth outcomes were not significantly associated with any of the outcomes [61,64].

Two studies tested multivariate associations between knowledge, attitudes, and behaviours potentially linked with sexual risk-taking. Of these, two found that adolescents who reported alcohol-drinking were more likely to be sexually active and less likely to use condoms [52,76], but no statistically significant association between drinking alcohol and reporting multiple sexual partners [76]. STI prevention knowledge were not significantly associated with unprotected sex [76].

#### Relationship factors

Relationship-level factors tested by three studies included having a child with one’s husband, living with a partner (compared to at home with parents/ caregivers), and gender-based violence [51,61,87]. HIV-positive adolescents and youth were more likely to report unintended pregnancy if they had biological children with their husband [61]. They were more likely to report multiple sexual partners and unintended pregnancies if they lived with their partner [87] or experienced gender-based violence [61,87].

#### Family and community factors

Four studies tested associations between four family and community factors associated with one or more sexual risk practices [52,54,63,64] using multivariate analyses, including: living with at least one biological parent, orphanhood, and parenting relationship (monitoring), and having a supportive family. Ugandan adolescents living alone were more likely to be sexually active in one study [52]. All other factors were not significantly associated with sexual risk-taking.

#### Structural factors

Six studies (five cross-sectional and one intervention) tested the effects of six structural factors or provisions on sexual risk-taking using multivariate analyses: poverty, food insecurity, participant employment, education (adolescent and mother), accessing grants and receiving livelihood training.

Employed adolescents were more likely to have ever had sex even when taking age into account [52]. Food insecurity was strongly associated with unintended pregnancies in the baseline sample of an RCT for HIV-positive orphaned and out-of-school adolescent girls in Zimbabwe [87]. In the same study, accessing grants in combination with health services and vocational training was associated with increased condom use and reduced transactional sex, but no reduction in multiple sexual partners was documented [78]. Findings on access to education were inconsistent with maternal education and poverty not associated with sexual risk-taking.

#### HIV-related factors

Four studies tested associations between fourteen HIV-related factors and sexual risk-taking practices using multivariate analyses. Knowing one’s own HIV-positive status and access to HIV support groups were associated with reduced unprotected sex. Findings on mode of infection and knowledge of partner’s HIV status were inconsistent [54,64,76]. Time since HIV diagnosis, time on ART, ART adherence, reporting opportunistic infections, disclosing HIV-status to partners, ART use/ access, and receiving ART care at a hospital were not significantly associated with unprotected sex [54,64].

### Interventions addressing sexual risk-taking among HIV-positive adolescents and youth

This review located eight studies of interventions aiming to address one or more sexual risk practices among HIV-positive adolescents or youth [88–93], but data on intervention effects were only available for four interventions, which are included in this review [40–42,78]. Three of the included intervention studies were individual-level randomised controlled trials [41,42,78] (Table 5), with one pre- and post-test experimental design study [40]. All interventions measured at least one high-risk sexual practice, with all measuring condom use or unprotected sex, three measuring number of sexual partners, and one measuring transactional sex. One of the studies reported effects on a composite sexual behaviour transmission score [42]. All four studies reported increases in condom use following the intervention; however, in one study the increase was not significant when intervention and control groups were compared post-intervention [42].

**Table 5:** Summary of included intervention studies.

*Author Year*, *Country*	*Intervention name*, *content*, *delivery mode*	*Study design*	*Follow-up time*	*Sample*^1^	*SQC score*^2^	*Outcomes*	*Results*
Lightfoot 2007 [41], Uganda	Cognitive Based Therapy (CBT) One-on-one with nurses 18 sessions	RCT Intervention: 50 Control: 50 Retention rate: 90%	Assessed at baseline and 3 months	14–21 year olds 72% female Not reported	75%	Number of sexual partners Consistent condom use Highly protected sex (abstinence or consistent condom use)	**Number of sexual partners:** Log number of sexual partners decreased (F 1,19 = 4.68, p = 0.04) **Consistent condom use:** Consistent condom use increased from 10% to 93% in intervention (p<0.01), control did not significantly change, from 15 to 12%. **Highly protected (abstinent or consistent condom use):** in intervention arm significantly increased from 74% to 98% (p<0.01), no change in control from 65% to 62%, NS.
Nhamo 2014 [78], Zimbabwe	Shaping the Health of Adolescents in Zimbabwe (SHAZ)-Plus! 0–6 months: both arms receive HIV/ SRH services + Life Skills education 7–12 months: *intervention* receives HIV/ SRH services + livelihood intervention (vocational training & grant); *control* receives only HIV/SRH services 13–18 months: both groups receive only HIV/ SRH services including testing	RCT pre- and post-test N = 710 Retention rate not reported	Baseline and follow-up at 6, 12, and 18 months; sexual outcomes reported for pre- test (baseline) to post-test (18 months)	16–19 years old 100% female Mode of infection not reported; orphaned and out of school, not pregnant at enrolment	66%	Multiple sexual partners Transactional sex Condom use	**Multiple sexual partners:** changed from 6% to 7%, OR = 1.05, .90–1.23, p = 0.504 **Transactional sex:** changed from 60% to 49%, OR = 0.87, 0.75–1.01, p = 0.067 **Condom use:** RR = 1.43 95%CI1.16–1.76, p<0.001
Senyonyi 2012 [42], Uganda	CBT Group counselling delivered by trained counsellors Recurring weekly sessions 80 min per session	RCT 328 contacted; 171 selected to participate in intervention N = 115 completed 3+ sessions: n = 80 intervention, n = 35 control	No information	12–18 years 53% female Vertically acquired HIV	55%	Sexual transmission score = number of sexual encounters (intercourse or penetrative sex), number of sexual partners, and unprotected penile penetrative vaginal sexual acts (intercourse; i.e., use of condoms, and continued abstinence)	**Sexual transmission behaviour score:** decrease in total score for both the intervention and control groups, Wilk’s λ = 0.951, F_1,113_ = 5.866, p = 0.017, partial η2 = 0.049. Repeated measures ANOVA showed no significant group differences at post-test, Wilk’s λ = 1.00, F(1,113) = 0.024, **p = 0.876**, partial η2 ≤ 0.001.
Snyder 2014 [40], South Africa	Hlanangani CBT Social Cognitive Theory Support groups delivered by lay counsellors 3 (2 hour) sessions over 11 months	Pilot study n = 109, 74 (68%) returning for all three sessions, analyses of	Start of session 1 (baseline) to end of session 3 (follow-up). Analyses n = 65 completers	16–24 years 95% female Unknown mode of infection Past-year diagnosis	41%	Condom use in previous 3 weeks (during intervention)	**Condom use:** increased by 12% (p = 0.049).

^1^ When available, age, gender, mode of infection, and other inclusion criteria are included.

^2^ Study Quality Checklist score.

Among the three interventions that had significant effects on reducing sexual risk-taking, one was an individual-based 18-session intervention delivered by nurses (n = 50 intervention and n = 50 control) [41], the second a group-based intervention focused on improving self-efficacy (n = 65) [40], and a third focused on addressing both individual and structural drivers of risk-taking through group-based life-skills training and livelihood support through grants and vocational training (n = 710) [78]. Though the first two studies are promising, the studies testing them reported on small sample sizes (n<120). To test their generalisability and scalability, these interventions need to be replicated with larger samples in real-life settings. The only large-scale intervention included in the review reported positive results from an intervention combining HIV/STI health services, life skills training, and livelihood components, such as small grants [78]. Findings from the combination intervention suggested that the results were due to the combination intervention rather than the life skills component alone [78]. This intervention focused on highly vulnerable HIV-positive adolescent girls, so additional research on whether these interventions may work among HIV-positive adolescents and youth in general would be beneficial.

## Discussion

This review includes 35 studies documenting the prevalence of sexual risk-taking, factors associated with high-risk sexual practices, and interventions for reducing sexual risk-taking in HIV-positive adolescents and youth from 13 sub-Saharan African countries. All studies reported on prevalence of high-risk sexual practices, and sixteen reported on at least one potential factor associated with sexual risk-taking. Four studies reported on interventions to reduce sexual risk-taking among HIV-positive adolescents. This section summarises the implications of the quality of included studies, followed by recommendations for a research agenda on the sexual practices of HIV-positive adolescents and youth.

### Quality of included studies

Most studies scored <50% in the Study Quality Checklist due to low scores on whether studies could establish causality. Notably, this was due to more than half the studies recruiting HIV-positive adolescents and youth as part of larger community-based samples, and not conducting analyses on the HIV-positive sub-sample, as this was not part of the original study aims. These datasets represent a unique opportunity to conduct secondary data analyses of the HIV-positive sub-samples in these studies to provide additional–more generalizable–insights from community-based samples. Most of the included studies focused on HIV-positive adolescents and youth recruited from facility-based samples. By engaging mostly adolescents who are already receiving care and support, the included studies are likely to under-estimate the prevalence of sexual risk-taking, as adolescents who are not engaged in care are more likely to experience poor sexual health outcomes [94]. A further limitation of the quality of included studies is that the majority of studies reported on cross-sectional analyses, which limited our ability to reach any conclusions about the causality and strength of the reported relationships between hypothesised factors and high-risk sexual practices. Rigorous longitudinal research is needed to better understand and test pathways of increased sexual risk-taking among HIV-positive adolescents and youth in sub-Saharan Africa.

Further, nearly half of the studies that tested hypothesised factors reported only on univariate analyses, mostly Chi square tests. A systematic review of condom use studies among adolescents in the United States also found that the majority of reviewed studies utilised univariate rather than multivariate analyses [95]. Theory- and policy-driven multivariate analyses are needed to gain a clearer picture of factors associated with sexual risk taking in HIV-positive adolescents and youth and how they are influenced by confounding factors. Quasi-experimental analyses can then be applied to assess which modifiable factors can be changed to reduce sexual risk-taking in this highly vulnerable population [96,97]. The poor quality of the studies and limited analytic techniques utilised may also explain why most of the reported findings were inconsistent.

### Implications for future research and practice

None of the included studies assessed longitudinal predictors, making it difficult to inform the design of large-scale policies and programming for HIV-positive adolescents and youth. However, the findings of this review have several implications for policy and practice.

First, HIV-positive adolescents and youth are reporting high levels of sexual risk-taking, which could lead to passing the virus to uninfected partners and children. These rates are high even though most of the included HIV-positive adolescents and youth were already in treatment and care. This suggests that improved sexual and reproductive health services for HIV-positive adolescents should be provided as part of regular HIV treatment and care. Successful models of integrating sexual and reproductive health services with HIV treatment and care should be designed, tested, and rolled out. Some adolescent- and youth-friendly models applied in resource-poor settings from organizations such as the Paediatric AIDS Treatment for Africa and its collaborators [98] should be further explored.

Second, nearly three-quarters of all pregnancies among HIV-positive adolescent women were reported to be unintended [53,74,78], higher than reported rates of unintended pregnancies among adolescent girls in South Africa [99]. While unintended pregnancy is not a risk practice itself, being pregnant during adolescence for HIV-positive adolescents presents a two-fold risk. On the one hand, adolescent pregnancy and motherhood has been linked to poorer health, educational, and socio-economic outcomes for both mother and child [99–102]. On the other hand, children of HIV-positive adolescent mothers are at increased risk of mother-to-child-transmission, due to poor adherence to ART and retention in care [14]. Reducing unintended pregnancies can have great positive effects in improving maternal and child health outcomes, particularly among HIV-positive adolescents [103]. The prevalence of unintended pregnancies among HIV-positive adolescents and youth suggest that the unmet contraceptive need among this population is even higher. Potential barriers to accessing contraception include quality of sexual and reproductive health services received at health facilities [25], stigma and discrimination [26,94], and lack of integration of family planning and HIV care services [104]. Additional research on how HIV-positive adolescent girls/ youth experience antenatal care services, PMTCT, and early parenthood is urgently needed as this cohort comes of age and enters their reproductive years.

Third, HIV-positive adolescents who are most vulnerable may also be at highest risk for onwards HIV transmission. Although most associations reported in the included studies were inconsistent, this review’s findings suggest that policies and programming must take into consideration the unique needs and profile of HIV-positive adolescents. In nine studies, sexual health outcomes were associated with other sexual risk-taking practices. For example, unintended pregnancy was associated with having multiple sexual partners. Only one intervention aimed to reduce multiple risk-taking, with no significant results between intervention and control groups [42]. Better understanding of factors shaping the syndemic of risk-taking among HIV-positive adolescents and youth will be important to help healthcare providers screen for and support those adolescents most at risk. Practitioners should be sensitised and trained to deal with the specific needs of HIV-positive adolescents and youth, as they initiate sexual and romantic relationships, and enter their reproductive years.

Fourth, the evidence on potential interventions, although based primarily on small-scale trials, suggests that improved non-medical care, psychosocial care and support, may reduce sexual risk-taking among HIV-positive adolescents. Testing the scalability of low-cost interventions delivered by lay counsellors such as support groups will be key to evidence-based programming for HIV-positive adolescents. HIV-specific support groups have had encouraging results in improving HIV treatment outcomes among adolescents and youth in other parts of South Africa [40,105], in neighbouring countries [106,107], as well as in this sample [108].

Finally, the results of the largest randomised controlled trial included in this review (n = 710) suggest that combinations of interventions may have a stronger impact on reducing sexual risk-taking among HIV-positive adolescents and youth [78]. Similarly to primary HIV prevention efforts, potential combinations of behavioural, biomedical and structural interventions should be tested [109,110]. Complex multi-level interventions require comprehensive financial, capital, and human resources to be designed and conducted. Therefore, testing combinations of existing low-resource real-life interventions, potentially using pragmatic research methodologies such as quasi-experimental designs, should precede setting up and testing potential combinations of interventions through randomised controlled trials.

Based on the quality of the evidence base, additional foundational and intervention research is needed to inform interventions for HIV positive teens. Evidence-based policy and programming could benefit from additional research with representative samples of HIV-positive adolescents and youth. The following research gaps were identified through this systematic review:

**High-risk sexual practices and exposure to violence:** This review has highlighted several gaps in research. First, better understanding of adolescents who are at risk of engaging in multiple high-risk sexual practices is pivotal to improved care and support programming. Second, although this systematic review did not focus on adolescent experiences of violence such as forced sex, gender-based violence, and domestic violence, multiple of the included studies found high rates of forced sexual initiation and gender-based violence amongst HIV-positive adolescents [74,75,87]. In light of the growing evidence linking exposure to violence and abuse among adolescents with poor health outcomes [111,112], it is important to understand whether and how violence exposure is associated with high-risk sexual practices in HIV-positive adolescents.

**Under-studied potential factors shaping sexual risk-taking:** A recently updated systematic review reports that vertically infected HIV-positive adolescents experience significant neurocognitive delays [18]. These findings are confirmed from research in South Africa, which has documented significant brain damage due to vertical HIV infection [113]. However, none of the included studies recorded whether the HIV-positive participants had neurocognitive problems, which may shape how they engage in sexual relationships. Only one study reported on mental health outcomes, controlling for sexual health outcomes [114], with data from a second study testing whether depression or anxiety was associated with sexual risk-taking. Further research is needed to elucidate how HIV-specific mental health issues affect sexual practices among HIV-positive adolescents and youth.

Mode of infection may be an important determinant of risk taking, with horizontally infected adolescents continuing to engage in higher risk-taking following HIV infection. Most of the studies in this review that reported mode of infection recruited primarily vertically infected adolescents. Though mode of infection is often difficult to ascertain and record [115], future studies should attempt to document factors that could ascertain the participants’ mode of infection, such as time on treatment, age at HIV diagnosis, and maternal orphanhood [10]. Additional research is needed on longitudinal predictors and interventions for horizontally infected adolescents and youth. Few studies reported age-disaggregated data, which would help providers to better understand how to tailor services and programmes to different age groups and to adapt to the changing needs of growing HIV-positive adolescent populations.

Studies of older adolescents and youth recorded several key relationship factors, such as whether the HIV-positive participant lived with their partners, had children with their husband vs. boyfriend, wanted additional children, or had disclosed their status in the relationship. However, most of the findings on these relationship topics were inconsistent. As HIV-positive adolescents get older and navigate sexual and romantic relationships, additional research is needed to understand how HIV-positive status, its disclosure and other relationship-related factors interact to shape sexual and reproductive outcomes [43,116,117].

Four studies [31,60,67,118] recruited a comparison sample of HIV-negative or HIV-status-unknown adolescents or youth, with three documenting how HIV-status knowledge was associated with sexual health outcomes [31,60,118]. Though this comparison was beyond the scope of this review, HIV-positive status was associated with increased protective sexual practices in one study and increased risk-taking in two other studies. These findings confirm those of a similar review which included studies from North America and Europe [30] but did not report consistent associations between knowledge of HIV-positive status and sexual practices. Findings from a nationally representative community-based study in South Africa suggest that knowledge of HIV-status, whether positive or negative, is strongly associated with reduced sexual risk-taking [29]. Further analyses on the effect of knowing one’s HIV-positive status on sexual risk-taking and the mechanisms through which disclosure shapes sexual practices is needed, particularly in light of the UNAIDS Fast Track target of 90% of HIV-positive adolescents learning their status by 2020 [7].

**Intervention research gaps:** First, although results from three cognitive-based therapy trials were encouraging, they need to be tested in larger-scale trials. Second, the majority of the studies in this review (17 of 35) took place in Uganda, including three of the four interventions. Additional quantitative and intervention research is needed on secondary HIV prevention in other sub-Saharan countries, particularly South Africa, Nigeria, and Kenya—home to the largest populations of HIV-positive adolescents in the region [5]. Third, several family-level factors were associated with reduced risk-taking in cross-sectional univariate analyses [52,59,63,74]. Although adolescence is a time for exploring and testing boundaries [119], family-based interventions may be key to support with difficult HIV-related issues such as disclosure, transition in care, and accessing prevention-of-mother-to-child treatment [120,121]. Fourth, in-depth analyses are needed of linkages between healthcare experiences (quality of services, access to services such as family planning, counselling and support groups) of HIV-positive adolescents and youth and their sexual and reproductive practices. Evidence to date is limited and inconsistent on the role of group-based care and support, though promising evidence from several programmes has been documented in recent conferences and workshops [105–107,122].

### Review limitations

In addition to the research gaps identified above, this review had several limitations. First, it included multiple outcomes to measure sexual risk-taking. Although evidence on linkages between sexually transmitted infections and high risk practices in adolescents are established [32,123,124], evidence that this review’s outcomes are associated with secondary HIV transmission is limited. Second, studies varied widely in terms of sample size, sampling strategies, and exact definitions of outcome measures, and the majority of studies were cross-sectional. Therefore, a meta-analysis was not possible, and our ability to reach conclusions on the prevalence and factors of sexual risk-taking among HIV-positive adolescents and youth was limited. Third, the analyses reported by the included studies were mostly univariate with actual statistics often not reported and confidence intervals missing, which resulted in a low quality of included evidence. Finally, the majority of studies were conducted in Uganda, including mostly HIV-positive adolescents and youth in care. Therefore, the results are not generalizable across the whole HIV-positive adolescent and youth population in sub-Saharan Africa. The evidence presented here must be interpreted with these methodological limitations in mind.

### Conclusion

HIV-positive adolescents have been neglected in HIV prevention efforts in the region, with few studies testing interventions aimed at supporting HIV-positive adolescents to reduce sexual and onwards vertical transmission (secondary prevention). Very few studies have rigorously documented potential risk and protective factors associated with increased secondary HIV-transmission risk. Longitudinal research is needed to establish and test emerging patterns between HIV-transmission risk and socio-demographic, HIV-specific, relationship, family, and structural-level factors. To address the potential for onwards HIV transmission, evidence is urgently needed on the effectiveness and feasibility of low-cost interventions to reduce HIV transmission from adolescents, both vertically and horizontally infected.

## Supporting information

S1 PRISMA Checklist PRISMA checklist. (DOC)

S1 File Data extraction form. (DOCX)

S2 File Study quality checklist and risk assessment bias. (DOCX)

S1 Table Study inclusion and exclusion criteria. (DOCX)

S2 Table Search string for databases searched in OvidSP. (DOCX)

S3 Table Search strings for PubMed & Proquest. (DOCX)

S4 Table Search strings for other smaller databases. (DOCX)

S5 Table Study screening checklist. (DOCX)

S6 Table Results of factors associated with sexual risk-taking among HIV-positive adolescents and youth by study. (DOCX)

S7 Table Prevalence of sexual risk-exposure outcomes reported by included studies. (DOCX)

S8 Table Results of the risk of bias assessments for all included studies. (DOCX)
