# Supplementary material for: Sex in the shadow of HIV: A systematic review of prevalence, risk factors, and interventions to reduce sexual risk-taking among HIV-positive adolescents and youth in sub-Saharan Africa
Source: PLoS One. 2017 Jun 5;12(6):e0178106. doi: 10.1371/journal.pone.0178106 (PMC5459342; doi:10.1371/journal.pone.0178106)
Supplement: S1 PRISMA Checklist — (DOC) [file pone.0178106.s001.doc]

| **Section/topic** | **#** | **Checklist item** | **Reported on page #** |
| --- | --- | --- | --- |
| **TITLE** | | |  |
| Title | 1 | Sex in the shadow of HIV: A systematic review of prevalence, risk factors, and interventions to reduce sexual risk-taking among HIV-positive adolescents and youth in Sub-Saharan Africa | 1 |
| **ABSTRACT** | | |  |
| Structured summary | 2 | **Background:** Evidence on sexual risk-taking among HIV-positive adolescents and youth in sub-Saharan Africa is urgently needed. This systematic review synthesizes the extant research on prevalence, factors associated with, and interventions to reduce sexual risk-taking among HIV-positive adolescents and youth in sub-Saharan Africa.  **Methods:** Studies were located through electronic databases, grey literature, reference harvesting, and contact with researchers. Preferred Reporting Items for Systematic Reviews and Meta-Analyses guidelines were followed. Quantitative studies that reported on HIV-positive participants (10-24 year olds), included data on at least one of eight outcomes (early sexual debut, inconsistent condom use, older partner, transactional sex, multiple sexual partners, sex while intoxicated, sexually transmitted infections, and pregnancy), and were conducted in sub-Saharan Africa were included. Two authors piloted all processes, screened studies, extracted data independently, and resolved any discrepancies. Due to variance in reported rates and factors associated with sexual risk-taking, meta-analyses were not conducted.  **Results:** 610 potentially relevant titles/abstracts resulted in the full text review of 251 records. Forty-two records (n=35 studies) reported one or multiple sexual practices for 13,536 HIV-positive adolescents/youth from 13 sub-Saharan African countries. Seventeen cross-sectional studies reported on individual, relationship, family, structural, and HIV-related factors associated with sexual risk-taking. However, the majority of the findings were inconsistent across studies, and most studies scored <50% in the quality checklist. Living with a partner, living alone, gender-based violence, food insecurity, and employment were correlated with increased sexual risk-taking, while knowledge of own HIV-positive status and accessing HIV support groups were associated with reduced sexual risk-taking. Of the four intervention studies (three RCTs), three evaluated group-based interventions, and one evaluated an individual-focused combination intervention. Three of the interventions were effective at reducing sexual risk-taking, with one reporting no difference between the intervention and control groups.  **Conclusion:** Sexual risk-taking among HIV-positive adolescents and youth is high, with inconclusive evidence on potential determinants. Few known studies test secondary HIV-prevention interventions for HIV-positive youth. Effective and feasible low-cost interventions to reduce risk are urgently needed for this group. | 2 |
| **INTRODUCTION** | | |  |
| Rationale | 3 | With increased access to antiretroviral treatment in sub-Saharan Africa, the number of children vertically infected with HIV who survive to adolescence has risen (1,2). Coupled with sustained high HIV-incidence among youth in the region, this has resulted in nearly 1.7 million HIV-positive adolescents (10-19 years old) in sub-Saharan Africa, with girls representing nearly two-thirds of this total (3–5). Despite global reductions in HIV prevalence, rates of new HIV infections remain the highest among 15-24 year old youth in sub-Saharan Africa (6). As their numbers continue to grow, adolescents and youth living with HIV are an essential group for secondary HIV prevention efforts (7).  HIV-positive adolescents and youth are at risk of passing on the virus to their sexual partners and children (8,9). They are additionally vulnerable to potential re-infection by HIV and more vulnerable to other sexually transmitted infections (STIs) compared to their HIV-negative peers (10). Adolescents are more likely than adults or younger children to adhere poorly to their medication (11–13) and in particular to treatment regimens to prevent mother-to-child-transmission (14). Low adherence and retention in care rates are strongly associated with resistance to available antiretroviral therapies, including second-line treatment when available (15,16). With limited access to second and third-line antiretroviral treatment, HIV-positive adolescents risk running out of treatment options or infecting others with resistant strains of the virus. In addition, HIV-positive adolescents experience a range of vulnerabilities that reduce the efficacy of generalised prevention programmes, including cognitive and mental health issues (17,18), family-related challenges (19,20), and material deprivation (21,22). Adolescents living with HIV in sub-Saharan Africa are particularly vulnerable to these risks due to poor access to healthcare services such as family planning, HIV testing, and treatment (23–27).  A small number of studies on adolescents living with HIV in sub-Saharan Africa report high rates of unprotected sex (28–30); however, little is known about rates of other high-risk practices, such as transactional sex, sex with older partners, and multiple concurrent sexual partners (31). In the general adolescent population, these high-risk sexual practices have been associated with higher odds of becoming infected with HIV (32). Though the evidence on different high-risk sexual practices among HIV-positive adolescents is nascent, understanding factors associated with sexual risk-taking is crucial for intervention development.  Although some interventions to reduce sexual risk behaviours have been conducted among HIV-positive adolescents in the United States (33–37), there is a dearth of research and interventions on secondary prevention among HIV-positive adolescents in the developing world (38). A 2010 WHO review of behavioural interventions for HIV positive prevention in middle and lower-income countries found 19 studies, none of which focused on young people (39). A recent review of sexual and reproductive health and rights interventions for youth living with HIV in sub-Saharan Africa located six small-scale interventions (38), only three of which quantitatively measured change in a sexual risk behaviour (40–42). | 3-4 |
| Objectives | 4 | The scope of this review is to assess the state of the evidence for three research questions:  1. What is the prevalence of sexual risk-taking among HIV-positive adolescents and youth in sub-Saharan Africa?  2. What factors (correlates, risk factors, or predictors) are associated with sexual risk-taking among HIV-positive adolescents and youth in sub-Saharan Africa?  3. Which interventions, aimed at reducing sexual risk-taking among HIV-positive adolescents and youth in sub-Saharan Africa, have been tested, and how effective were they? | 4-5 |
| **METHODS** | | |  |
| Protocol and registration | 5 | Study protocol (PROSPERO registration number CRD42015025871). | 6 |
| Eligibility criteria | 6 | **Inclusion criteria** applied consisted of study population, design, sampling strategy, outcome measures, population type, and language (S1 Table). To document outcome prevalence and factors associated with the outcomes, cross-sectional surveys and longitudinal prospective cohort studies were included. Although Randomised Controlled Trials (RCT) provide the strongest form of evidence about intervention impact, due to the small number of RCTs identified in preliminary searches, this review also included studies with less rigorous designs: pre-post intervention comparisons and post-intervention comparisons with ‘control’ populations. Studies measuring at least one of eight high-risk sexual practices either as a primary or secondary outcome were included. High-risk sexual practices included early sexual debut, unprotected sex (inconsistent condom use/ contraception use), having an older partner, transactional sex, having multiple sexual partners, sex whilst intoxicated, sexually transmitted infections, and unwanted adolescent pregnancies, or a composite measure of two of these outcomes – as defined by each study. Reports in English and French were reviewed to allow for publications from Western and Central Africa. | 5 |
| Information sources | 7 | **Search Strategy:** In September-November 2015, the first author searched the online databases of PsycARTICLES, Embase, Global Health, MEDLINE, and PsycINFO, PubMed, CINAHL, ProQuest, and WHO Afro Library, the Cochrane and Campbell databases and the PROSPERO register of systematic reviews. The first author also searched International AIDS Society conference abstracts and presentations, as well as websites of major international and regional organisations, such as the World Health Organization (WHO), Joint UN Program for HIV/AIDS (UNAIDS), the UN Children’s Fund (UNICEF), United States Agency for International Development (USAID), UN Family Planning Agency (UNFPA), International Planned Parenthood Federation (IPPF), and Population Council. Key search terms for sample population (children, adolescents, teenagers and youth), all high-risk sexual practices, location (sub-Saharan Africa) and timeline were included (S2-S4 Tables). All searches were conducted within the publication date limits of 1983 or the closest date limit available, reflecting the time since HIV has been diagnosed in adolescents and youth. | 5-6 |
| Search | 8 | Presented in tables in supplementary materials. | S2-S4 tables |
| Study selection | 9 | **Screening:** The screening process followed the Cochrane Collaboration Handbook guidelines (45). Following merging and de-duplication, two authors reviewed titles and abstracts for relevance. When available, full-text documents were retrieved and checked for eligibility against inclusion and exclusion criteria (S1 Table), and a set of pre-agreed screening questions (S5 Table). Email requests for clarifications, unpublished data, and data from published studies were sent to researchers working on sexual risk-taking of HIV-positive adolescents and youth. Recent guidance on systematic reviews suggests that there is a potential bias from including studies with very small samples in systematic reviews (46). To minimise this bias, when studies reported ≥50 HIV-positive adolescents and youth, but age-disaggregated data was not available in the included reports, authors were contacted for age-disaggregated data for 10-24 year old HIV-positive participants. If additional data were provided, the studies were included in the review. Reference lists of the included studies and of other relevant reviews were screened for further eligible titles. | 6 |
| Data collection process | 10 | **Data extraction:** Data was extracted from full-text records by the first author (ET) using a pre-piloted data extraction form (S1 File). A second independent reviewer checked the data extraction for each included study (MP/FM/RH) and any discrepancies were resolved through discussion. Records reporting analyses from the same dataset were checked for data duplication, with the largest sample taken if multiple reports were available for the same outcome measure. | 6-7 |
| Data items | 11 | For longitudinal studies reporting a change in an outcome of interest, baseline values of the reported outcome were extracted as prevalence. If data was not reported for HIV-positive adolescents or youth specifically but authors provided the raw data, the prevalence for sexual risk-taking was calculated for HIV-positive adolescent or youth, via frequencies in SPSS. In such instances, the same definition of the sexual risk outcome as the primary study was used. For example, Viegas and colleagues reported rates of early sexual debut defined as ‘before the age of 18’ for a sero-assorted sample (47). Using a dataset shared by the research team, this review’s first author computed the prevalence of ‘sexual debut before 18 years old’ for HIV-positive youth. Where relevant, the prevalence of risky sexual practice was computed based on the prevalence of related safe sexual practices reported. For example, if a study reported condom use at last intercourse as 40%, the rate of unprotected sex at last intercourse was computed as 60%. Both reported and computed prevalence of inconsistent condom use/ unprotected sex are reported. | 7 |
| Risk of bias in individual studies | 12 | Risk of bias across studies was assessed using a Study Quality Checklist and risk assessment form (S2 File). The form drew from guidance on assessing systematic bias from the Cochrane Handbook for experimental designs (randomised controlled trials (RCTs), non-randomised controlled trials and pre- and post-test experimental design) (45), and the Cambridge Quality Checklist for systematic reviews of risk factors (48). The checklist was adapted in line with a systematic review of internalised stigma among people living with HIV (49). Adaptation included assessing sampling strategies at two levels: facility/ community and individual level, and assessing each individual association between potential factors and the outcome of interest. For each potential determinant, each outcome-determinant relationship was scored as a percentage of the total score possible from the Study Quality Checklist (SQC). SQC scores for each outcome-predictor relationship are reported in S6 Table. | 8 |
| Summary measures | 13 | Three measures were used:   1. Prevalence (%) 2. Odds ratios or Chi square for risk factors 3. Between group differences for RCTs/ trials | 8 |
| Synthesis of results | 14 | Data synthesis: Given the diversity of primary studies and outcomes measured, and the cross-sectional nature of the majority of the included studies, a meta-analysis was not conducted, in order to avoid potentially misleading conclusions (48). To reflect the diversity of reported prevalence rates, data was reported as the range of reported values for studies using the same definitions for each outcome. | 8 |

Page 1 of 2

| **Section/topic** | **#** | **Checklist item** | **Reported on page #** |
| --- | --- | --- | --- |
| Risk of bias across studies | 15 | Risk of bias across studies was assessed using a Study Quality Checklist and risk assessment form (S2 File). The form drew from guidance on assessing systematic bias from the Cochrane Handbook for experimental designs (randomised controlled trials (RCTs), non-randomised controlled trials and pre- and post-test experimental design) (45), and the Cambridge Quality Checklist for systematic reviews of risk factors (48). The checklist was adapted in line with a systematic review of internalised stigma among people living with HIV (49). Adaptation included assessing sampling strategies at two levels: facility/ community and individual level, and assessing each individual association between potential factors and the outcome of interest. For each potential determinant, each outcome-determinant relationship was scored as a percentage of the total score possible from the Study Quality Checklist (SQC). SQC scores for each outcome-predictor relationship are reported in S6 Table. | 7 |
| Additional analyses | 16 | N/A |  |
| **RESULTS** | | |  |
| Study selection | 17 | Results from different database searches were merged, resulting in 3,314 records. Grey literature searches and hand-searches of references of included studies resulted in an additional 61 records. After de-duplication, two authors (ET/KK) reviewed 610 titles and abstracts and the full text documents for 251 results. A total of 42 records were included in this systematic review, which reported data from k=35 studies (Table 2). | 8 |
| Study characteristics | 18 | **Study design:** The 35 included studies reported data from N=13,536 HIV-positive adolescents and youth living in 13 countries. Four studies described interventions evaluated through RCTs (k=3) or pre- and post-test experimental design (k=1), and the remaining k=31 reported on cross-sectional data.  **Participant characteristics:** Participants were mostly female (k=35 studies: 47%-100%), vertically infected (k=9 studies: 43%-100%), and on ART (k=9 studies: 0%-88%). Of the 17 studies that reported whether HIV-positive adolescents and youth knew their status, the majority recruited only adolescents who knew their status (k=13). In the six studies which recorded disclosure of HIV status to others, just under half of HIV-positive adolescents had shared their HIV-positive status with their partners (31%-74%) (50–55).  **Outcome measures:** Thirty-three studies assessed sexual practices of HIV-positive adolescents as the primary outcome, and two reported them as secondary outcomes. The outcomes measured varied in terms of the recall periods of measurement and exact definitions (Table 3). Nine studies reported on only one sexual practice, while the rest reported on two or more sexual practices. The most common definitions for each reported outcome were: (1) sex before 15 years old (k=3), (2) sex before 18 years old (k=3), (3) inconsistent/no condom use at last sexual encounter (k=14), (4) current use of modern contraception (k=6), (5) having an older partner at first sexual intercourse (k=4), (6) having ever had transactional sex (k=3), (7) multiple sexual partners in the past 12 months (k=5), (8) sex while intoxicated (k=2), (9) ever having had an STI or STI symptoms (k=5), and (10) ever been pregnant (k=7). All outcomes were based on self-reports, except for two studies reporting results of STI tests for Hepatitis B (HBV) (56) and Human Papillomavirus (HPV) (57). One study reported on a composite sexual risk-taking score (42). In addition to the above high-risk sexual practices, three studies reported on risk-exposure sexual outcomes such as forced sex, non-consensual first sex, gender-based violence, as their main outcomes. These outcomes were beyond the scope of the initial study protocol, therefore information on sexual risk-exposure outcomes is provided in S7 Table. | 8-9 |
| Risk of bias within studies | 19 | **Quality assessment of included studies**  Of the seventeen studies that reported on potential risk factors or intervention effects, most scored below 50% in the Study Quality Checklist (k=14, range 10%-75%, S6 Table). The reasons for the low scores included study design and analyses, sampling strategies, response/retention rates, and sample size, which are discussed in this section.  Study design: Of the included studies sixteen were cross-sectional, three were prospective cohorts , one was an experimental pre- and post-test , and seven were RCTs. The three prospective cohort studies did not report analyses of change, nor did they assess longitudinal predictors of sexual risk-taking; hence, only relevant baseline data was extracted on prevalence and potential associated factors. Data from four RCTs reported only on prevalence or factors associated with the outcomes of interest using cross-sectional data from the baseline of the study. The included data on prevalence and potential factors associated with sexual risk-taking were cross-sectional (k=31), while intervention data was based on a pre- and post-test experimental study (k=1) and three RCTs (k=3).  Sampling: Sampling strategies were assessed at two levels: community/clinics and individual level (Table 2). Of the 31 observational studies, seventeen recruited only from healthcare facilities, primarily through purposefully selected facilities (k=14) (50,53,56–67). Most of these studies recruited only HIV-positive participants (k=11). Seven other studies recruited only from communities through random or stratified sampling (29,68–73). Most of these studies recruited HIV-positive participants as part of larger community-based samples. Five studies recruited participants through combined facility/community sampling (31,54,55,74,75).  At the individual-level sampling, eleven studies recruited through total or random sampling at each study site (29,31,32,50,54,68–71,73,76), eight recruited through purposeful sampling (47,52,55,56,58,61–63), and another eight through convenience sampling (28,53,57,59,60,66,74,75). The three intervention studies which reported sampling data recruited through a combination of purposeful and convenience sampling at both the facility/community and individual levels (40–42).  Sample sizes: The included studies reported on n=13,536 HIV-positive adolescents and youth (10-24 years old), with sample sizes ranging between n=65 and n=1,703. Fourteen of thirty-five studies had a sample size smaller than n<400 participants, which was chosen as the cut-off for a study powered to detect predictors and intervention effects, based on a recent systematic review which assessed studies of predictors of internalised HIV-stigma (49). Studies with sample sizes <400 scored lower in the Study Quality Checklist.  Response and retention rates: Most studies (k=26) did not report response or retention rates or did not have response rates for the HIV-positive sub-population, making it difficult to assess the extent of selection bias. Of studies that reported response or retention rates (k=9), the majority stated retention of ≥90% (41,42,53,54,60,74,75,77), with only one reporting a retention rate of 89.6% (28). Two of the three small-scale intervention studies analysed only data from completers, who accounted for 67.3% (42) and 59.6% (40) of those who enrolled in the studies.  Strength of associations between factors/ interventions and outcomes: In the cross-sectional data analyses, 13 studies conducted univariate analyses (such as Chi square tests, univariate logistic regressions, or Student’s t-tests) and eleven reported on multivariate analyses (such as logistic regressions, multivariate log binomial regression, random effects logit model estimations) of associations between potential factors and the outcomes, controlling for potential confounders (S6 Table). All four experimental design studies reported within-group change for at least one sexual behaviour over time, with three randomising participants to a control and an intervention group (41,42,78). | 10 |
| Results of individual studies | 20 | Results are presented in three sections: (1) Prevalence of sexual risk-taking (Table 3, S6), (2) Factors associated with sexual risk-taking among HIV-positive adolescents and youth (Table 4), (3) Interventions addressing sexual risk-taking among HIV-positive adolescents and youth.  Within ‘Factors associated with sexual-risk taking reported in included studies’, results were grouped under: (1) Individual-level factors, (2) Relationship factors, (3) Family and community factors, (4) Structural factors, and (5) HIV-related factors. | 10-12 |
| Synthesis of results | 21 | Given the diversity of primary studies and outcomes measured, and the cross-sectional nature of the majority of the included studies, a meta-analysis was not conducted, in order to avoid potentially misleading conclusions (48). | 12-18 |
| Risk of bias across studies | 22 | Risk of bias assessment presented in table S8. | S8 table |
| Additional analysis | 23 | Given the diversity of primary studies and outcomes measured, and the cross-sectional nature of the majority of the included studies, a meta-analysis was not conducted, in order to avoid potentially misleading conclusions (48). | 12-18 |
| **DISCUSSION** | | |  |
| Summary of evidence | 24 | This review includes 35 studies documenting the prevalence of sexual risk-taking, factors associated with high-risk sexual practices, and interventions for reducing sexual risk-taking in HIV-positive adolescents and youth from 13 sub-Saharan African countries. All studies reported on prevalence of high-risk sexual practices, and sixteen reported on at least one potential factor associated with sexual risk-taking. Four studies reported on interventions to reduce sexual risk-taking among HIV-positive adolescents. This section summarises the implications of the quality of included studies, followed by recommendations for a research agenda on the sexual practices of HIV-positive adolescents and youth. | 19-24 |
| Limitations | 25 | In addition to the research gaps identified above, this review had several limitations. First, it included multiple outcomes to measure sexual risk-taking. Although evidence on linkages between sexually transmitted infections and high risk practices in adolescents are established (32,123,124), evidence that this review’s outcomes are associated with secondary HIV transmission is limited. Second, studies varied widely in terms of sample size, sampling strategies, and exact definitions of outcome measures, and the majority of studies were cross-sectional. Therefore, a meta-analysis was not possible, and our ability to reach conclusions on the prevalence and factors of sexual risk-taking among HIV-positive adolescents and youth was limited. Third, the analyses reported by the included studies were mostly univariate with actual statistics often not reported and confidence intervals missing, which resulted in a low quality of included evidence. Finally, the majority of studies were conducted in Uganda, including mostly HIV-positive adolescents and youth in care. Therefore, the results are not generalizable across the whole HIV-positive adolescent and youth population in sub-Saharan Africa. The evidence presented here must be interpreted with these methodological limitations in mind. | 29 |
| Conclusions | 26 | HIV-positive adolescents have been neglected in HIV prevention efforts in the region, with few studies testing interventions aimed at supporting HIV-positive adolescents to reduce sexual and onwards vertical transmission (secondary prevention). Very few studies have rigorously documented potential risk and protective factors associated with increased secondary HIV-transmission risk. Longitudinal research is needed to establish and test emerging patterns between HIV-transmission risk and socio-demographic, HIV-specific, relationship, family, and structural-level factors. To address the potential for onwards HIV transmission, evidence is urgently needed on the effectiveness and feasibility of low-cost interventions to reduce HIV transmission from adolescents, both vertically and horizontally infected. |  |
| **FUNDING** | | |  |
| Funding | 27 | ET, MP, and KK were supported by the Evidence for HIV Prevention in Southern Africa [MM/EHPSA/UCT/05150014]. Additional support for MP and ET was provided by the joint Green Templeton-Clarendon Scholarship fund. RH was supported by the Economic and Social Research Council (ESRC) Doctoral Training Centre Studentship and the Clarendon-Jesus College Old Members' Fund. FM and LC were supported by the European Research Council (ERC) under the European Union’s Seventh Framework Program [FP7/2007-2013]/ERC grant agreement n° 313421, University of Oxford's ESRC Impact Acceleration Account (Grant 1311-KEA-004 & 1609-GCRF-227), and the Philip Leverhulme Trust [PLP-2014-095 AQ6]. The funders had no role in study design, data collection and analysis, decision to publish, or preparation of the manuscript. | Financial statement for PONE |

*From:*  Moher D, Liberati A, Tetzlaff J, Altman DG, The PRISMA Group (2009). Preferred Reporting Items for Systematic Reviews and Meta-Analyses: The PRISMA Statement. PLoS Med 6(7): e1000097. doi:10.1371/journal.pmed1000097

For more information, visit: **www.prisma-statement.org**.

Page 2 of 2
