# Supplementary material for: Sex in the shadow of HIV: A systematic review of prevalence, risk factors, and interventions to reduce sexual risk-taking among HIV-positive adolescents and youth in sub-Saharan Africa
Source: PLoS One. 2017 Jun 5;12(6):e0178106. doi: 10.1371/journal.pone.0178106 (PMC5459342; doi:10.1371/journal.pone.0178106)
Supplement: S1 File — (DOCX) [file pone.0178106.s002.docx]

**S1 File. Data extraction form**

**Notes:**

- Complete this form only for included studies.
- Be consistent in the order and style you use to describe the information for each report.
- Record any missing information as unclear or not described, to make it clear that the information was not found in the study report(s), not that you forgot to extract it.

1. General Information

| Study ID *(surname of first author and year first full report of study was published e.g. Smith 2001)* |  |
| --- | --- |
| Date form completed *(dd/mm/yyyy)* |  |
| Name of person extracting data | ET |
| Study author contact details |  |
| Publication type *(e.g. full report, abstract, presentation, grey literature)* | peer-reviewed article  conference abstract  published report  conference presentation  unpublished report  other ________________________ |
| Notes: | |

1. Characteristics of included studies

*Methods*

|  | **Descriptions as stated in report/paper** |  |
| --- | --- | --- |
| **Outcome measures** | Primary outcomes:   1. Early sexual debut 2. Unprotected sex 3. Contraception use 4. Sex with an older partner 5. Transactional sex 6. Multiple partners 7. Sex drunk or on drugs 8. Sexually Transmitted Infections 9. Unwanted pregnancy 10. Combination of any of the above   Secondary outcomes:  Any of the above outcomes  Other: |  |
|  | Reliability coefficient or convergent validity coefficient |  |
| **Study design** | Study without a comparison group – No analysis of change  Inadequately controlled study – No analysis of change  Study without a comparison group – With analysis of change  Inadequately controlled study – With analysis of change  Controlled non-experimental study – No analysis of change  Controlled non-experimental study – with analysis of change  Randomized experiment – Targeting a risk factor |  |
| **Unit of observation** | individual  dyad (couple/ parent-child)  other | |
| ***Types of intervention**^[[1]](#footnote-1)^ | Behavioural  Biomedical  Structural  Social protection  Combination of interventions |  |
|  | ***If combination of interventions, please describe the intervention:*** | |
| **Comparison** | multiple arms/ comparison groups  another intervention  treatment as usual  no comparison  other | |
|  | ***Please describe comparison group/ arms:*** | |
| ***Duration of intervention** |  | |
| **Duration of participation** | From recruitment to last follow-up: _________ months  Follow-up duration: ________ months *(from intervention to follow-up)*  ***Notes on follow-up:*** | |

*Participants*

|  | Description | Details |
| --- | --- | --- |
| Population of interest | - HIV-positive adolescents (10-19 year old) only - HIV-positive youth (15-24 years old) - Some HIV-positive adolescents or youth included in the overall sample |  |
| Demographics/ characteristics of population of interest | Mode of infection | Vertical ___________ %  Horizontal __________%  Not reported (NR) |
|  | Gender (Report gender composition for all arms) | female _____%  Not reported (NR) |
|  | Treatment status | ART naïve  On ART treatment  Special groups (defaulters, lost-to-follow-up, PMTCT patient)  NR |
|  | Disclosure | Know own status ______ %  NR  Disclosure to others: _____ %  NR |
| Setting and context | Location |  |
|  | Community or facility (clinic) sampling | community-based sampling  health facility-based sampling  NR |
|  | Sampling of individual participants | total population or random sampling  purposive sampling  convenience sampling  NR |
| Sampling | Inclusion criteria |  |
|  | Exclusion criteria |  |
|  | Method of recruitment of participants *(e.g. phone, mail, clinic patients)* |  |
|  | Sample size |  |
|  | *Cluster-RCTs – Inter cluster correlation | _______ % NR |
|  | Non-RCTs – Response rate | _______ %  NR |
|  | *RCTs – Retention rate  – differential attrition | _____ % NR  _____ % NR |

*Results*

| Results – overview | Baseline imbalances *(if applicable)* |  |
| --- | --- | --- |
|  | Withdrawals and exclusions (%) |  |
|  | Analysis type | ITT  Completers  Not reported |
|  | Missing data (rate and analysis method) |  |
|  | Confounding factors/ effect modifiers accounted for |  |

**OUTCOMES**

| **Outcome** | **Prevalence** | **Crude (specify, e.g. OR, RR, IRR and specify the reference group)** | **Adjusted (specify, e.g. OR, RR, IRR and specify the reference group)** | **Intervention effect (if relevant)** | **Notes** |
| --- | --- | --- | --- | --- | --- |
|  |  |  |  |  |  |
|  |  |  |  |  |  |
|  |  |  |  |  |  |
|  |  |  |  |  |  |

**DETERMINANTS**

| **Determinant** | **Prevalence (N=?)** | **Outcome 1** | | | | **Outcome 2** | | | |
| --- | --- | --- | --- | --- | --- | --- | --- | --- | --- |
|  |  | **Relationship to outcome (delete as appropriate)** | **Crude (specify, e.g. OR, RR, IRR and specify the reference group)** | **Adjusted (specify, e.g. OR, RR, IRR and specify the reference group)** | **Notes** | **Relationship to outcome (delete as appropriate)** | **Crude (specify, e.g. OR, RR, IRR and specify the reference group)** | **Adjusted (specify, e.g. OR, RR, IRR and specify the reference group)** | **Notes** |
|  |  | correlate  risk factor  causal risk factor |  |  |  | correlate  risk factor  causal risk factor |  |  |  |
|  |  | correlate  risk factor  causal risk factor |  |  |  | correlate  risk factor  causal risk factor |  |  |  |

1. Fields marked with * are to be completed for intervention trials only. [↑](#footnote-ref-1)
