# Supplementary material for: Sex in the shadow of HIV: A systematic review of prevalence, risk factors, and interventions to reduce sexual risk-taking among HIV-positive adolescents and youth in sub-Saharan Africa
Source: PLoS One. 2017 Jun 5;12(6):e0178106. doi: 10.1371/journal.pone.0178106 (PMC5459342; doi:10.1371/journal.pone.0178106)
Supplement: S2 File — (DOCX) [file pone.0178106.s003.docx]

## **S2 File. Study Quality Checklist**

| Quality Assessment^[[1]](#footnote-1)^ | |  |
| --- | --- | --- |
| **Study design score**^[[2]](#footnote-2)^ | | |
| 1 | Study without a comparison group – No analysis of change | |
| 2 | Inadequately controlled study – No analysis of change | |
| 3 | Study without a comparison group – With analysis of change | |
| 4 | Inadequately controlled study – With analysis of change | |
| 5 | Controlled non-experimental study – No analysis of change | |
| 6 | Controlled non-experimental study – With analysis of change | |
| 7 | Randomised experiment targeting a risk factor | |
| **Cluster sampling (cluster= facility, community, school or other)** | | |
| 3 | Total population or random sampling | |
| 2 | Purposive sampling | |
| 1 | Convenience sampling | |
| 0 | Not reported | |
| **Within-cluster sampling of participants** | | |
| 3 | Total population or random sampling | |
| 2 | Purposive sampling | |
| 1 | Convenience sampling | |
| 0 | Not reported | |
| **Response rates** | | |
| 2 | Response *or* retention rates ≥70% *or* differential attrition ≤10%^[[3]](#footnote-3)^ | |
| 1 | Response rate <70% *or* retention rate <70% *or* differential attrition >10% | |
| 0 | Not reported | |
| **Sample size score** | | |
| 1 | Sample size ≥400 (cohort studies) and ≥200 (experimental designs) | |
| 0 | Sample size <400 (cohort studies) and <200 (experimental designs) | |
| **Outcome measures (***complete for each different outcome***)** | | |
| 4 | Self-reported measure validated through biomarker | |
| 3 | Self-reported measure validated through other measurements | |
| 2 | Self-reported measure collected through special techniques to improve response rate (e.g. AUDIO-ACASI/ AMASI, ACASI/ AMASI, sealed envelopes, etc.) | |
| 1 | Self-reported measure collected through regular techniques | |
| 0 | Not reported | |
| **Determinant measures (***complete for each different determinant***)** | | |
| 3 | Reliability coefficient ≥.75 and reasonable face validity *or* criterion or convergent validity coefficient ≥ 0.3 *or* more than one instrument or information source used | |
| 2 | Use of an instrument used in previous studies with the same sample | |
| 1 | Use of a non-validated measure | |
| 0 | Not reported | |
| **Determinant (causal risk factor/ risk factor) score (***complete for each different determinant***)** | | |
| 6 | Analysis with variation in the predictor and adequately balanced, with analysis of change | |
| 5 | Analysis with variation in the predictor and adequately balanced, no analysis of change | |
| 4 | Analysis with variation in the predictor but inadequately balanced, with analysis of change | |
| 3 | Analysis without variation in the predictor, with analysis of change | |
| 2 | Analysis with variation in the predictor but inadequately balanced, no analysis of change | |
| 1 | Analysis without variation in the predictor, no analysis of change | |

***Risk of Bias Assessment***

| Risk of Bias criteria ↓/ Study Design 🡪 | Randomised controlled trials | Non-randomised controlled trials, pre-and post-test, experimental designs | Non-experimental longitudinal or cross-sectional designs | Notes |
| --- | --- | --- | --- | --- |
| Random sequence generation (selection bias) | low risk  high risk  not clear | high risk  low risk  not clear | high risk  low risk  not clear | For non-RCTs, consider sampling strategy. |
| Allocation concealment (selection bias) | low risk  high risk  not clear | high risk  low risk  not clear | high risk  low risk  not clear |  |
| Blinding of participants and personnel (performance bias) | low risk  high risk  not clear | high risk  low risk  not clear | high risk  low risk  not clear |  |
| Blinding of outcome assessment (outcome bias) | low risk  high risk  not clear | high risk  low risk  not clear | high risk  low risk  not clear | For non-RCTs, consider outcome measurements methods. |
| Incomplete outcome data (attrition bias) | low risk  high risk  not clear | high risk  low risk  not clear | high risk  low risk  not clear | For non-RCTs, consider reporting of (1) missing data, (2) response rate, and (3) retention rate. |
| Selective reporting (reporting bias) | low risk  high risk  not clear | high risk  low risk  not clear | high risk  low risk  not clear |  |
| Other bias | low risk  high risk  not clear | high risk  low risk  not clear | high risk  low risk  not clear | Contamination in cluster RCTs. |

1. Scoring to be completed separately for each analyzed association between an outcome and an associated factor. [↑](#footnote-ref-1)
2. Items 1 and 2 will be scored for cross-sectional study designs. [↑](#footnote-ref-2)
3. Response rate scored for cross-sectional study designs. Retention rate scored for longitudinal study design*s.* [↑](#footnote-ref-3)
