# Supplementary material for: Sex in the shadow of HIV: A systematic review of prevalence, risk factors, and interventions to reduce sexual risk-taking among HIV-positive adolescents and youth in sub-Saharan Africa
Source: PLoS One. 2017 Jun 5;12(6):e0178106. doi: 10.1371/journal.pone.0178106 (PMC5459342; doi:10.1371/journal.pone.0178106)
Supplement: S1 Table — (DOCX) [file pone.0178106.s004.docx]

**S1 Table. Study inclusion and exclusion criteria**

| Topic | Inclusion criteria | Exclusion criteria |
| --- | --- | --- |
| Study population | - HIV-positive - Any of the sample in the 10-24 year old range | HIV-exposed adolescents or youth |
| Study design | - Intervention studies: randomised control trials, pilot studies, pre-post intervention studies (controlled and not). - Quantitative or mixed methods reports of longitudinal cohort studies. - Cross-sectional studies | - Qualitative studies - Case-control studies |
| Sampling | - Located in sub-Saharan Africa - Single or multi-country - Random or non-random sampling - Community and facility-based sampling | Sub-Saharan African immigrants living outside of sub-Saharan Africa |
| Outcome measure | - Any of the sexual behaviours listed above or a combination of these sexual behaviours, either as primary or secondary outcome; - Biological markers: STI test results and pregnancy. | - Combination risk methods where data on individual sexual behaviours are not reported - Studies reporting proxy behaviours, intentions, knowledge, or awareness related to the outcomes of interest. |
| Publication type | - Peer-reviewed journal article - Grey literature - Conference abstracts and presentations - Dissertations | - Conference abstracts without additional information |
| Language | - English - French - Portuguese - Spanish | - Any other language |
