# Supplementary material for: Sex in the shadow of HIV: A systematic review of prevalence, risk factors, and interventions to reduce sexual risk-taking among HIV-positive adolescents and youth in sub-Saharan Africa
Source: PLoS One. 2017 Jun 5;12(6):e0178106. doi: 10.1371/journal.pone.0178106 (PMC5459342; doi:10.1371/journal.pone.0178106)
Supplement: S2 Table — (DOCX) [file pone.0178106.s005.docx]

**S2 Table. Search string for databases searched in OvidSP (PsychARTICLES, Embase, Global Health, Ovid MEDLINE and PsycINFO)^[[1]](#footnote-1)^**

| 1 | Population | Adolescents or youth living with HIV | (((HIV or AIDS or ((human or acquired) adj1 (immunodeficiency or immune-deficiency or immuno-deficiency))) adj2 (child* or adolescen* or teen* or you*)) or ALHIV or PHIV or BHIV).ab,ti,hw. |
| --- | --- | --- | --- |
| 2 | Outcome(s) | Early sexual debut | (early adj1 sexual adj1 (debut or initiation)).ab,hw,ti. Or |
| 3 |  | Unprotected sex | (((safe or unsafe or protected or unprotected) adj1 (sex or intercourse)) or abstinen*).ab,hw,ti. |
| 4 |  | Contraception use | (condom or IUD or implant* or contracepti* or hormon* or inject* or spermicide or diaphragm or (dual adj1 (protection or method))).ab,hw,ti. |
| 5 |  | Sex with an older partner | (((old* or age$disparate or intergenerational) adj1 sex* adj1 partner)).ab,hw,ti. |
| 6 |  | Transactional sex | (((sugar adj1 (daddy or daddies)) or ((transactional or survival) adj1 sex))).ab,ti,hw. |
| 7 |  | Multiple partners | (MCP or ((multiple or concurrent) adj2 partner*)).ab,hw,ti. |
| 8 |  | Sex drunk or on drugs | ((sex adj1 drunk) or (sex adj2 drug*)).ab,hw,ti. |
| 9 |  | Sexually Transmitted Infections | (sexually transmitted infection* or STI* or chlamydia or gonorrh$ea or syphilis or (herpes simplex virus or HSV) or HPV).ab,hw,ti. |
| 10 |  | pregnancy | Pregnan*.ab,hw,ti. |
| 11 | Final outcome search string 2 OR 3 OR 4 OR 5 OR 6 OR 7 OR 8 OR 9 OR 10 | | |
| 12 | Location | Sub-Saharan Africa | ((sahara* adj1 africa) or Angola or Benin or Botswana or Burkina Faso or Burundi or Cameroon or Cape Verde or Central African Republic or Chad or Comoros or Congo or Brazzaville or Democratic Republic of Congo or Cote d'Ivoire or Djibouti or Equatorial Guinea or Eritrea or Ethiopia or Gabon or Gambia or Ghana or Guinea or Guinea$Bissau or Kenya or Lesotho or Liberia or Madagascar or Malawi or Mali or Mauritania or Mauritius or Mozambique or Namibia or Niger or Nigeria or (Reunion adj3 africa) or Rwanda or Sao Tome or Senegal or Seychelles or Sierra Leone or Somalia or South Africa or Sudan or Swaziland or Tanzania or Togo or Uganda or Western Sahara or Zambia or Zimbabwe).ab,cp,hw,ti. |
| 19 | FINAL SEARCH STRING 1 AND 11 AND 12 | | |

1. .ab. denotes a word in the abstract; hw. denotes head words; .pt. denotes a Publication Type term; .ti. denotes a word in the title. [↑](#footnote-ref-1)
