# Supplementary material for: Sex in the shadow of HIV: A systematic review of prevalence, risk factors, and interventions to reduce sexual risk-taking among HIV-positive adolescents and youth in sub-Saharan Africa
Source: PLoS One. 2017 Jun 5;12(6):e0178106. doi: 10.1371/journal.pone.0178106 (PMC5459342; doi:10.1371/journal.pone.0178106)
Supplement: S3 Table — (DOCX) [file pone.0178106.s006.docx]

**S3 Table. Search Strings for PubMed & Proquest**

| **Database** | **PubMed** | **Proquest** |
| --- | --- | --- |
| Search String | (((("HIV"[Mesh] OR "Acquired Immunodeficiency Syndrome"[Mesh] OR "HIV-1"[Mesh]) AND ("Child"[Mesh] OR "Adolescent"[Mesh] OR "Young Adult"[Mesh]) AND ("Sexual Behavior"[Mesh] OR "Sexual Partners"[Mesh] OR "Sexual Abstinence"[Mesh] OR "Social Behavior"[Mesh] OR "Reproductive Health"[Mesh] OR "Coitus"[Mesh] OR "Contraception"[Mesh] OR "Contraception, Postcoital"[Mesh] OR "Contraception, Barrier"[Mesh] OR "Contraception, Immunologic"[Mesh] OR "Contraception Behavior"[Mesh] OR "Sexually Transmitted Diseases/epidemiology"[Majr] OR "Sexually Transmitted Diseases/prevention and control"[Majr] OR "Sexually Transmitted Diseases/psychology"[Majr] OR "Pregnancy in Adolescence"[Mesh] OR "Pregnancy"[Mesh] OR "Pregnancy, Unplanned"[Mesh] OR "Pregnancy, Unwanted"[Mesh]) AND ("Randomized Controlled Trial"[Publication Type] OR "Non-Randomized Controlled Trials as Topic"[Mesh] OR "Multicenter Studies as Topic"[Mesh] OR "Cross-Over Studies"[Mesh] OR "Evaluation Studies as Topic"[Mesh] OR "Program Evaluation"[Mesh] OR "Evaluation Studies"[Publication Type] OR "Pilot Projects"[Mesh] OR "Cohort Studies"[Mesh] OR "Lost to Follow-Up"[Mesh] OR "Epidemiologic Studies"[Mesh] OR "Odds Ratio"[Mesh]) AND ("Africa"[Mesh] OR "Africa South of the Sahara"[Mesh] OR "South Africa"[Mesh] OR "Africa, Western"[Mesh] OR "Africa, Southern"[Mesh] OR "Africa, Eastern"[Mesh] OR "Africa, Central"[Mesh]) | (((HIV OR AIDS OR ((human OR acquired) adj1 (immunodeficiency OR immunodeficiency OR immunodeficiency))) adj2 (child* OR adolescent* OR teen* OR you*)) OR ALHIV OR PHIV OR BHIV) AND ((early adj1 sexual adj1 (debut OR initiation)) OR ((safe OR unsafe OR protected OR unprotected) adj1 (sex OR intercourse)) OR abstinen* OR (condom OR IUD OR implant* OR contraception* OR hormone* OR inject* OR spermicide OR diaphragm OR (dual adj1 (protection OR method))) OR ((old* OR age disparate OR intergenerational) adj1 sex* adj1 partner) OR (sugar adj1 (daddy OR daddies)) OR ((transactional OR survival) adj1 sex) OR (MCP OR (multiple or concurrent) adj2 partner*) OR ((sex adj1 drunk) OR (sex adj2 drug*)) OR (sexually transmitted infection* OR STI* OR chlamydia OR gonorrhoea OR gonorrhea OR syphilis OR (herpes simplex virus OR HSV) OR HPV) OR Pregnan*)  AND ((sahara* adj1 africa) OR Angola OR Benin OR Botswana OR Burkina Faso OR Burundi OR Cameroon OR Cape Verde OR Central African Republic OR Chad OR Comoros OR Congo OR Brazzaville OR Democratic Republic of Congo OR Cote d'Ivoire OR Djibouti OR Equatorial guinea OR Eritrea OR Ethiopia OR Gabon OR Gambia OR Ghana OR guinea OR guinea bissau OR Kenya OR Lesotho OR Liberia OR Madagascar OR Malawi OR Mali OR Mauritania OR Mauritius OR Mozambique OR Namibia OR Niger OR Nigeria OR (Reunion adj3 africa) OR Rwanda OR Sao Tome OR Senegal OR Seychelles OR Sierra Leone OR Somalia OR South Africa OR Sudan OR Swaziland OR Tanzania OR Togo OR Uganda OR Western Sahara OR Zambia OR Zimbabwe) |
| Publication Types | n/a | AND stype.exact("Conference Papers & Proceedings" OR "Scholarly Journals" OR "Dissertations & Theses" OR "Working Papers" OR "Government & Official Publications") |
| Language | AND (English[lang] OR French[lang] OR Spanish[lang] OR Portuguese[lang]) | AND la.exact("English" OR "Portuguese" OR "French" OR "Spanish") |
| Timeline | AND (("1983/01/01"[PDAT] : "3000/12/31"[PDAT]) | AND pd(>19830101) |
