# Supplementary material for: Sex in the shadow of HIV: A systematic review of prevalence, risk factors, and interventions to reduce sexual risk-taking among HIV-positive adolescents and youth in sub-Saharan Africa
Source: PLoS One. 2017 Jun 5;12(6):e0178106. doi: 10.1371/journal.pone.0178106 (PMC5459342; doi:10.1371/journal.pone.0178106)
Supplement: S4 Table — (DOCX) [file pone.0178106.s007.docx]

**S4 Table. Search strings for other smaller databases (PopLine, WHO Africa, and CINAHL)**

| **Search terms** | | | | **Popline** | **WHO Africa** | **CINAHL** |
| --- | --- | --- | --- | --- | --- | --- |
| 1 | Population | Adolescents or youth living with HIV | | Title: (((HIV or AIDS) AND (child* OR adolescen* OR teen* OR you*)) OR ALHIV OR BHIV OR PHIV)  OR  Keyword: (((HIV or AIDS) AND (child* OR adolescen* OR teen* OR you*)) OR ALHIV OR BHIV OR PHIV) | (HIV OR AIDS) AND (child OR adolescent OR teenager OR youth) | ((HIV or AIDS) AND (child* OR adolescen* OR teen* OR you*)) OR ALHIV OR BHIV OR PHIV) |
| 2 | Outcome(s) | Early sexual debut  Unprotected sex  Contraception use  Sex with an older partner  Transactional sex  Multiple partners  Sex drunk or on drugs  Sexually Transmitted Infections  Unwanted pregnancy | | N/A | sex | (((early N1 sexual N1 (debut or initiation)) OR (((safe or unsafe or protected or unprotected) N1 (sex or intercourse)) or abstinen*) Or (condom or IUD or implant* or contracepti* or hormon* or spermicide or diaphragm or (dual N1 (protection or method))) Or (((old* or age$disparate or intergenerational) N1 sex* N1 partner)) Or (((sugar N1 (daddy or daddies)) or ((transactional or survival) N1 sex))) OR ((MCP or (multiple or concurrent) N2 partner*)) OR ((sex N1 drunk) or (sex N2 drug*)) OR (sexually transmitted infection* or STI* or chlamydia or gonorr$ea or syphilis or (herpes simplex virus or HSV) or HPV) OR Pregnan*) |
| 3 | Location | Sub-Saharan Africa | | (sahara* and africa) or (south* and Africa) or (east* or Africa) | N/A | selected Africa under ‘Geographic Subset’ |
| Final search | | | 1 AND 3 | | 1 AND 2 | 1 and 2 |
