# Supplementary material for: Sex in the shadow of HIV: A systematic review of prevalence, risk factors, and interventions to reduce sexual risk-taking among HIV-positive adolescents and youth in sub-Saharan Africa
Source: PLoS One. 2017 Jun 5;12(6):e0178106. doi: 10.1371/journal.pone.0178106 (PMC5459342; doi:10.1371/journal.pone.0178106)
Supplement: S5 Table — (DOCX) [file pone.0178106.s008.docx]

**Table S5. Study screening questions**

*Note: continue to subsequent question only if the answer to the current question is “yes”*. *Otherwise exclude study from the review.*

| **Study Characteristics** | **Eligibility criteria** | **Eligibility criteria met?** | | |
| --- | --- | --- | --- | --- |
|  |  | **Yes** | **No** | **Unclear** |
| Participants and setting | Adolescents and youth (10-24 years old) |  |  |  |
| Location of study | Study samples in at least one country in sub-Saharan Africa |  |  |  |
| Type of study | Experimental study including randomized controlled trials (RCTs) or cluster-randomized trials (CRTs). |  |  |  |
|  | Quasi-experimental studies including quasi-randomized trials, pre-and-post studies, and interrupted time series studies (ITSs). |  |  |  |
|  | Observational longitudinal cohort studies |  |  |  |
|  | Observational cross-sectional study |  |  |  |
| Types of outcome measures | Primary outcome:   1. Early sexual debut 2. Unprotected sex 3. Contraception use 4. Sex with an older partner 5. Transactional sex 6. Multiple partners 7. Sex drunk or on drugs 8. Sexually Transmitted Infections 9. Unwanted pregnancy 10. Combination of any of the above   Secondary outcomes:  Any of the above outcomes |  |  |  |
| INCLUDE | Reason for exclusion: | | | |
