# Supplementary material for: Sex in the shadow of HIV: A systematic review of prevalence, risk factors, and interventions to reduce sexual risk-taking among HIV-positive adolescents and youth in sub-Saharan Africa
Source: PLoS One. 2017 Jun 5;12(6):e0178106. doi: 10.1371/journal.pone.0178106 (PMC5459342; doi:10.1371/journal.pone.0178106)
Supplement: S6 Table — (DOCX) [file pone.0178106.s009.docx]

**Table S6. Results of correlates of sexual risk-taking among HIV-positive adolescents and youth by study**

| *First author, year* | *Outcome* | *SCQ score^[[1]](#footnote-1)^* | *Factors: correlates or predictors*  *(test results and significance, NR – not reported, NS – not significant)^[[2]](#footnote-2)^* |
| --- | --- | --- | --- |
| Ankunda 2011 [77]; Ankunda 2016 [50] | Condom use | 38% | **Individual:** current contraceptive use (PRR=4.07, 95%CI 1.62-85.78) |
|  |  | 38% | **Family:** desiring a child (PRR=0.44, 95%CI 0.21-0.90) |
| Baryamutuma 2010 [59] | Early sexual debut | 10% | **Structural:** out of school (chi sq NR, p<0.001) |
|  | Condom use | 10% | **Relationship:** single (chi sq NR, p<0.05) |
|  | Older sexual partner | 10% | **Individual:** females (chi sq NR, p=0.008) |
|  | STI risk | 10% | **Sexual practices:** older sexual partner (chi sq NR, p<0.001) |
|  | Pregnancy | 10% | **Family:** not living with parents (chi sq NR, p<0.001) |
| Birungi 2009 [51]; Birungi 2009 [74]; Obare 2010 [118] | Contraception use at first sex | 41% | **Individual:** older age more use (chi sq, p<0.05 among HIV+ only) |
|  |  | 41% | **Individual:** sex (NS) |
|  |  | 41% | **Family**: lives with at least one biological parent (NS) |
|  |  | 41% | **Family**: no siblings (compared to any siblings) (chi sq NR, p<0.05) |
|  |  | 41% | **Structural**: attend school (NS) |
|  |  | 41% | **HIV**: knows HIV+ status (NS in mixed sample_ |
|  |  | 41% | **HIV**: discordant relationship (CQC 41%) (45% vs. 65% in concordant, NS) |
|  | Contraception use in current/ past relationship | 41% | **Individual:** older age (chi sq, p<0.05) |
|  |  | 41% | **Individual**: male gender (chi sq, p<0.05) |
|  |  | 41% | **Individual**: attends school (NS) |
|  |  | 41% | **Family**: lives with at least one biological parent (NS) |
|  |  | 41% | **Family**: lives with siblings (NS) |
|  |  | 41% | **Relationship**: married/lives with partner (-0.55 (0.19),p<0.001) |
| Birungi 2011 [61] | Post-partum contraception | 34% | **Individual:** study site (OR=0.5, 95%CI 0.2-0.9, p<0.05) |
|  |  | 34% | **Individual**: poor birth outcomes (NS) |
|  |  | 34% | **Relationship:** paternity husband vs. boyfriend/fiancé (NS) |
|  |  | 34% | **Structural**: maternal education (NS) |
|  |  | 34% | **Sexual practices**: pregnancy order (NS) |
|  |  | 34% | **Sexual practices**: age at 1^st^ pregnancy (NS) |
|  |  | 34% | **Sexual practices**: unintended pregnancy (NS) |
|  | Unintended pregnancy | 34% | **Individual:** study site (NS) |
|  |  | 34% | **Relationship**: paternity husband vs. boyfriend/fiancé (OR=0.1, 95%CI 0.1-0.2), p<0.001) |
|  |  | 34% | **Structural**: maternal education (NS) |
|  |  | 34% | **Sexual practices:** pregnancy order (NS) |
|  |  | 34% | **Sexual practices**: age at 1^st^ pregnancy (NS) |
| Holub 2010 [63] | Unprotected sex/ protected sex/ no sex | 24% | **Individual:** age (NS) |
|  |  | 24% | **Individual**: gender (NS) |
|  |  | 24% | **Individual**: religious guidance (NS) |
|  |  | 28% | **Family**: # parents lost (NS) |
|  |  | 31% | **Family:** parental monitoring (NS) |
|  |  | 24% | **Family**: social support (NS) |
|  |  | 24% | **Family**: monitoring*support (OR=1.65, 95%CI 0.98-2.79, p=0.06); aOR=1.57, 95%CI 0.91-2.75, p=0.11 |
|  |  | 24% | **Structural**: currently in school (NS) |
|  |  | 24% | **HIV:** knows HIV status (NS) |
|  |  | 24% | **HIV**: time since diagnosis less than one year, more unprotected sex (chi sq, p=0.03) |
|  |  | 24% | **Sexual practices**: ever had transactional sex (NS) |
| Kaggwa 2012 [125] | Condom use at last sex | 24% | **Individual:** age 19-21 year old vs. 16-18 year old (OR=0.27, p<0.01) |
|  |  | 24% | **Individual**: age 22-24 year old vs. 16-18 year old (NS) |
|  |  | 24% | **Individual**: clinically depressed (OR=0.68, p<0.05) |
|  |  | 24% | **Individual**: anxiety—low compared to moderate (NS) or high (NS) |
|  |  | 24% | **Family/community**: social support (NS) |
|  |  | 24% | **HIV**: mode of infection (NS) |
|  |  | 24% | **HIV**: ARV use (NS) |
|  | >1 partner in past 12 months | 24% | **Individual:** Age 19-21 year old vs. 16-18 year old (NS) |
|  |  | 24% | **Individual**: age 22-24 year old vs. 16-18 year old (NS) |
|  |  | 24% | **Individual**: clinically depressed (NS) |
|  |  | 24% | **Individual**: anxiety—low compared to moderate (NS) or high (NS) |
|  |  | 24% | **Family/community**: social support (NS) |
|  |  | 24% | **HIV**: mode of infection (NS) |
|  |  | 24% | **HIV**: ARV use (NS) |
| Katusiime 2012 [56] | HBV infection | 41% | **HIV:** CD4+ count >250 (chi sq, p<0.001) |
|  |  | 28% | **HIV**: WHO stage I or II (chi sq, p<0.001) |
| Lightfoot 2007 [41] | Consistent condom use | 75% | **Intervention:** access to healthcare services 🡪 one-on-one nurse-led counselling (NR, p<0.01) |
|  | Number of sexual partners | 75% | **Intervention:** access to healthcare services 🡪 one-on-one nurse-led counselling (F 1,19=4.68, p=0.04) |
|  | Highly protected sex | 75% | **Intervention:** access to healthcare services 🡪 one-on-one nurse-led counselling (NR, p<0.01) |
| Mbalinda 2015 [52]; Mbalinda 2015 [114] | Ever had sex | 38% | **Individual:** age 15-19 (CQC 38%) OR=6.28, 2.63-14.99, p<0.001 |
|  |  | 35% | **Individual**: female gender (chi sq, p<0.05) |
|  |  | 35% | **Individual**: being a volunteer/at home vs. student (chi sq, p<0.05) |
|  |  | 35% | **Individual**: education level (NS) |
|  |  | 35% | **Individual**: region (chi sq, p<0.05) |
|  |  | 38% | **Individual**: never drunk alcohol (OR=0.49, 95%CI 0.28-0.87, p=0.02) |
|  |  | 35% | **Individual**: ever smoked a cigarette (chi sq, p<0.05) |
|  |  | 35% | **Individual**: religion (NS) |
|  |  | 38% | **Family/community**: living alone (OR=4.33, 95%CI 1.13-16.62, p=0.03 |
|  |  | 35% | **Family/ Community:** had a friend who smoked (chi sq, p<0.05) |
|  |  | 35% | **Family/ Community:** friend who drunk (chi sq, p<0.05) |
|  |  | 38% | **Family/ Community:** influenced to drink/ smoke (chi sq, p<0.05) |
|  |  | 35% | **Structural:** in school (CQC 38%) OR=.20, 0.13-0.30, p<0.001 |
|  |  | 35% | **Structural:** education level (NS) |
|  |  | 35% | **Structural:** occupation 🡪 being a volunteer vs. student (OR7.28, 3.85-13.75, p<0.001) |
|  |  | 41% | **Structural:** occupation 🡪 at home vs. student (OR8.61, 5.15-14.41, p<0.001) |
|  |  | 41% | **HIV:** never STI treatment (OR=0.19, 95%CI 0.11-0.32, p<0.001) |
| Mhalu 2013 [76] | Sexual debut <=15 | 38% | **Individual:** female gender (chi sq=3.865, p=0.048) |
|  | Multiple sexual partners | 41% | **Individual:** older age (NS) |
|  |  | 41% | **Individual**: gender (NS) |
|  |  | 41% | **Individual**: not using alcohol (NS) |
|  |  | 41% | **Individual**: district of residence (NS) |
|  |  | 41% | **Individual**: STI prevention knowledge (NS) |
|  |  | 41% | **Family/community**: supportive family (NS) |
|  |  | 41% | **Structural**: education status (NS) |
|  |  | 41% | **HIV**: not knowing partner’s HIV status (OR=2.62, 95%CI 1.14-5.10, p=0.023) |
|  |  | 41% | **HIV:** ART use (OR=.38, 95%CI 0.17-0.85, p=0.018) |
|  |  | 41% | **Sexual practices**: sexual debut before 15 (NS) |
|  | Unprotected sex | 41% | **Individual:** younger age 15-19 (OR=2.76, 95%CI 1.05-7.27, p=0.040) |
|  |  | 41% | **Individual**: gender (NS) |
|  |  | 41% | **Individual:** not using alcohol (OR=0.40, 95%CI 0.17-0.84, p=0.017) |
|  |  | 41% | **Individual**: district of residence (NS) |
|  |  | 41% | **Individual**: STI prevention knowledge (NS) |
|  |  | 41% | **Family/community**: supportive family (NS) |
|  |  | 41% | **Structural**: education status (NS) |
|  |  | 41% | **HIV**: not knowing partner’s HIV status (NS) |
|  |  | 41% | **HIV**: ART use (NS) |
|  |  | 41% | **Sexual practices**: sexual debut before 15 (NS) |
| Nhamo 2013 [87] | Unintended pregnancy | 52% | **Relationship:** GBV (aRR=1.28, 95%CI 1.05-1.57) |
|  |  | 52% | **Sexual practices:** multiple sex partners (aRR=2.94, 95%CI 2.38-3.63) |
|  |  | 52% | **Sexual practices:** sex without a condom (aRR=1.83, 95%CI 1.48-2.26) |
|  |  | 52% | **Relationship:** being married/living with a partner (aRR=3.00, 95%CI 2.45-3.68) |
|  |  | 52% | **Structural:** food insecurity (aRR=1.68, 95%CI 1.51-2.30) |
| Nhamo 2014 [78] | Condom use | 66% | **Intervention - Structural:** grants + livelihood training + SRH services (RR=1.43, 95%CI 1.16-1.76, p<0.001) |
|  | Transactional sex | 66% | **Intervention - Structural:** grants + livelihood training + SRH services (OR=0.87, 95% CI 0.75-1.01, p0.067) |
|  | Multiple sexual partners | 66% | **Intervention - Structural:** grants + livelihood training + SRH services (NS) |
| Nöstlinger 2015 [55] | Ever had sex | 31% | **Individual:** study site: NS |
| Obare 2010 [53] | Condom use | 34% | **Individual:** male gender (chi sq. p<0.05) |
|  | Injectable contraception | 34% | **Individual:** female gender (chi sq, p<0.01) |
|  | Pregnant | 34% | **Individual:** female gender (chi sq, p<0.01) |
|  | Older partner currently | 34% | **Individual:** female gender (chi sq, p<0.01) |
|  | Older partner at first sex | 34% | **Individual:** female gender (chi sq, p<0.01) |
|  | Used contraception at first sex | 34% | **Individual:** gender (NS) |
|  | Ever used contraception | 34% | **Individual:** gender (NS) |
|  | Currently using contraception | 34% | **Individual:** gender (NS) |
| Senyonyi 2012 [42] | Sexual transmission behaviour score | 55% | **Intervention – Structural:** group counselling (Wilk’s λ = 0.951, F(1,113) = 5.866, p = 0.017, partial η2 = 0.049 for pre and post-test comparisons, but NS comparing intervention to control group). |
| Snyder 2014 [40] | Condom use at last sex | 41% | **Intervention – HIV:** support groups (+12%, p<0.01) |
| Test 2012 [75] | Partner 5+ years older | 31% | **Individual:** female gender (chi sq, p<0.01) |
|  | Partner 10+ years old | 31% | **Individual:** gender (NS) |
|  | Transactional sex | 31% | **Individual:** female gender (chi sq, p=0.03) |
|  | Condom use past 6 months | 31% | **Individual:** gender (NS) |
|  | Lifetime number of partners | 31% | **Individual:** gender (NS) |
|  | Transactional sex (among females only n=32) | 31% | **Individual:** age (NS) |
|  |  | 31% | **Individual**: median number of school years (NS) |
|  |  | 31% | **Individual**: works for wage (NS) |
|  |  | 31% | **Sexual practices:** median lifetime partners (chi sq, p=0.03) |
|  |  | 31% | **Sexual practices:** condom use inconsistent (NS) |
|  |  | 31% | **Sexual practices**: median age at sexual debut (NS) |
|  |  | 31% | **Sexual practices**: age asymmetry at first sex (NS) |
|  |  | 31% | **HIV**: sexual HIV acquisition (NS) |
| Toska 2015 [54] | Unprotected sex at last intercourse | 45% | **Individual:** age (NS) |
|  |  | 45% | **Individual**: female gender (NS) |
|  |  | 45% | **Family/community**: lives with biological caregiver (NS) |
|  |  | 45% | **Family/community**: orphanhood (NS) |
|  |  | 45% | **HIV:** knowledge of own HIV+ status (OR=4.36, 95%CI 1.08-17.5, p<0.05) |
|  |  | 45% | **HIV:** mode of infection (NR, p<0.05) |
|  |  | 45% | **HIV**: time on ART (NS) |
|  |  | 45% | **HIV**: ART adherence (NS) |
|  |  | 45% | **HIV**: knows partner HIV status (NS) |
|  |  | 45% | **HIV**: disclosed to partner (NS) |
|  |  | 45% | **HIV**: opportunistic infections (NS) |
|  |  | 45% | **HIV**: hospital care (NS) |
|  |  | 45% | **Sexual practices/outcomes**: STI symptoms (NS) |
| Viegas 2015 [47] | Sex before 18 | 25% | **Individual:** gender (NS) |
|  | Condom use at last sex | 25% | **Individual:** gender (NS) |
|  | Multiple sexual partners | 25% | **Individual:** gender (NS) |
|  | STIs | 25% | **Individual:** gender (NS) |

1. Percentage score of the quality of the association between the outcome and the correlate based on the Study Quality Checklist (SQC). [↑](#footnote-ref-1)
2. When multiple results were available from several studies, results of the most rigorous methodology or the largest sample size are reported here. [↑](#footnote-ref-2)
