# Supplementary material for: Sex in the shadow of HIV: A systematic review of prevalence, risk factors, and interventions to reduce sexual risk-taking among HIV-positive adolescents and youth in sub-Saharan Africa
Source: PLoS One. 2017 Jun 5;12(6):e0178106. doi: 10.1371/journal.pone.0178106 (PMC5459342; doi:10.1371/journal.pone.0178106)
Supplement: S7 Table — (DOCX) [file pone.0178106.s010.docx]

**Table S7. Prevalence rates of sexual risk-exposure outcomes reported by included studies**

| *Outcome* | *Definition* | *Studies* | *Rates)* | *Gender disaggregation* | *Notes* |
| --- | --- | --- | --- | --- | --- |
| Gender-based violence | Any self-reported experience of physical, sexual abuse or forced sex | Nhamo 2013 [87] | 34% | All female | 28% physical; 13% sexual; 7% forced sex |
| Forced sex | Had consensual first sex | Birungi 2009 [51]; Birungi 2009 [74] | 73% | 63% F, 89% M |  |
|  | Ever forced sex | Nhamo 2013 [87] | 7% | All female |  |
|  | Not clear | Test 2012 [75] | 22% | 29% F, 7% M |  |
