# Supplementary material for: Sex in the shadow of HIV: A systematic review of prevalence, risk factors, and interventions to reduce sexual risk-taking among HIV-positive adolescents and youth in sub-Saharan Africa
Source: PLoS One. 2017 Jun 5;12(6):e0178106. doi: 10.1371/journal.pone.0178106 (PMC5459342; doi:10.1371/journal.pone.0178106)
Supplement: S8 Table — (DOCX) [file pone.0178106.s011.docx]

**Table S8. Results of the risk of bias assessments for all included studies**

| *Study* | *Selection bias* | *Performance bias* | *Outcome bias* | *Attrition bias* | *Reporting bias* | *Other bias* |
| --- | --- | --- | --- | --- | --- | --- |
| Ankunda 2011 [77]; Ankunda 2016 [50] | low-high | not clear | high | not clear | high | not clear |
| Bakeera-Kitaka 2008 [58] | high | high | high | low | not clear | not clear |
| Banura 2008 [57] | high | low | low | low | low | not clear |
| Baryamutuma 2010 [59] | not clear | not clear | not clear | not clear | not clear | not clear |
| Beyeza-Kasheysa 2011 [60] | low | low | high | low | low | not clear |
| Birungi 2009 [51]; Birungi 2009 [74]; Obare 2010 [118] | low | low | high | low | low | not clear |
| Birungi 2011 [61] | high | high | high | low | low | not clear |
| Cataldo 2012 [28] | high | high | high | high | high | not clear |
| Gavin 2006 [68] | low | not clear | high | low | low | not clear |
| Gray 1998 [69] | low | low | low | low | low | low |
| Heffron 2010 [70] | low | not clear | not clear | high | low | low |
| Hendriksen 2007 [71]; Steffenson 2011 [83] | low | low | high | low | high | not clear |
| Hoffman 2008 [62] | low-high | low | low | not clear | low | not clear |
| Holub 2010 [63] | high | low | high | not clear | low | not clear |
| Kaggwa 2012 [125] | high | high | high | high | high | not clear |
| Katusiime 2012 [56] | high | low | low | not clear | high | not clear |
| Kembo 2012 [72] | low | low | low | low | low | not clear |
| Lightfoot 2007 [41] | low-high | high | low | low | low | not clear |
| Malaju 2013 [67] | low-high | high | high | low | low | not clear |
| Mbalinda 2015 [52]; Mbalinda 2015 [114] | high | high | high | not clear | low | not clear |
| Mhalu 2013 [76] | low | not clear | high | not clear | low | not clear |
| Morris 2012 [31] | low | low | high | not clear | high | not clear |
| Muyindike 2012 [65] | low-high | high | high | low | low | not clear |
| Nhamo 2013 [87]; Nhamo 2014 [78] | low | low-high | low-high | not clear | high | not clear |
| Nöstlinger 2015 [55] | high | high | high | not clear | high | not clear |
| Obare 2010 [53] | high | high | high | low | low | not clear |
| Pascoe 2015 [73] | low | low | low | low-high | low | not clear |
| Santelli 2013 [32] | low | high | high | high | low | not clear |
| Senyonyi 2012 [42] | high | high | high | high | low | not clear |
| Shisana 2014 [29] | low | low | high | high | low | not clear |
| Snyder 2014 [40] | high | high | high | high | low | not clear |
| Test 2012 [75] | high | high | high | low | low | not clear |
| Toska 2015 [54] | low | high | high | low | low | not clear |
| Viegas 2015 [47] | high | high | low | not clear | low | not clear |
| Wanyenze 2011 [66] | high | high | high | high | low | not clear |
